# How observing others influences the subsequent acquisition of fear of bodily sensations

**DOI:** 10.1016/j.xjmad.2026.100199

**Published:** 2026-08-04

**Authors:** Carlotta P. Albert, Christoph Benke, Christiane A. Melzig

**Affiliations:** a Marburg University, Institute of Psychology, Department of Clinical Psychology, Experimental Psychopathology and Psychotherapy, Marburg, Germany; b Center for Mind, Brain and Behavior (CMBB), Marburg, Giessen, and Darmstadt, Germany

**Keywords:** Observational learning, Vicarious learning, Learning history, Interoceptive threat, Fear conditioning, Panic disorder

## Abstract

Observational fear learning preceding a first-hand learning experience is assumed to potentially sensitize individuals, resulting in greater fear acquisition. In a laboratory setting, we therefore investigated how different demonstrator responses to temporary dyspnea influence consecutive direct fear acquisition. 74 participants were randomly assigned to three experimental groups. In the two observational learning groups, participants observed a demonstrator completing an interoceptive differential fear conditioning paradigm, during which one of two visual cues (conditioned stimuli, CS) was paired with temporary dyspnea for the demonstrator, displaying either a calm, non-fearful or a fearful response to the unconditioned stimulus (US). Participants of the control condition saw the similar setup with no demonstrator being present. Then, the three groups completed a differential direct fear conditioning paradigm with first-hand temporary dyspnea as the US. During observational learning, observers of any demonstrator, but not the control condition, acquired significant contingency knowledge, indicated by greater US-expectancy in response to CS + vs. CS -. During direct acquisition, participants of all groups showed significant fear learning, indicated by greater US-expectancy, skin conductance responses and startle response magnitudes to CS + vs. CS -. Although fear responses decreased in these measures, significant CS + /CS - differences were maintained upon the end of extinction. Evaluating the effect of the different demonstrators, exploratory analyses revealed that startle response potentiation to CS + vs. CS - was only present in the fearful demonstrator group during acquisition. Although preliminary in nature, this suggests that the acquisition of fear of interoceptive threat may be facilitated by preceding observational learning from a fearful demonstrator.

## Highlights

• We examined how prior observational learning influences direct fear acquisition.
• Contingency knowledge transferred from observational learning to direct acquisition.
• The influence of different demonstrators’ responses warrants further research..

## 1 Introduction

Along with own experiences, observing another person’s response to a certain stimulus or event is crucial for acquiring information about what is dangerous or safe and research has demonstrated that fear can be acquired just by the observation of a demonstrator [1]. Experimental studies revealed that observational learning from a demonstrator completing a conditioning paradigm during which one geometrical shape is paired with an electro-tactile shock leads to significant fear responses as indicated by fear reports, skin conductance responses (SCRs) or greater startle response magnitudes, when the observers were later exposed to the same geometrical shape [2], [3]. Further extending previous studies, a recent study conducted by Alcan, Benke, and Melzig [4] demonstrated that the expression of fear following observational learning is also evident when threat is coming from inside the body. Interoceptive threat can result from bodily sensations, such as palpitations or dyspnea, i.e., the shortness of breath. Specifically, temporary dyspnea has been identified as a potent unconditioned stimulus (US) for investigating associative fear learning involving interoceptive stimuli, also known as interoceptive fear conditioning [5]. Alcan et al. [4] found that observation of a demonstrator showing fearful responses to the experience of temporary dyspnea (observational US) during presentation of a geometrical shape was followed by increased fear and US-expectancy ratings as well as greater autonomic arousal and increased defensive reflex mobilization when the observers later viewed the same geometrical shape.

At the same time, it is also possible that information about the safety or harmlessness of a stimulus can be transmitted through observational learning. These processes have been described as positive modeling or observational safety learning referring to situations in which a demonstrator is observed while exhibiting a positive response to a stimulus or safety is inferred from complete absence of an aversive event for the demonstrator following the presentation of a stimulus [6], [7]. Initial studies examined this phenomenon in children by using pictures of novel animals that were paired with either happy or scared faces to model different observational learning experiences. These studies found preliminary support that fear beliefs towards unknown animals are reduced or remain unchanged, when they were first paired with happy faces [7]. However, observational learning experiences can be manifold and may also convey information that cannot easily be assigned to the binary categories of safety and danger. For example, it is conceivable that even observing a calm or non-fearful response of a demonstrator to a stimulus could result in less fear in the observers during later exposure to the same stimulus. This possibility may be of particular relevance in the context of interoceptive stimuli, as overtly positive or happy responses to inherently aversive bodily sensations would not necessarily be expected. Rather, a calm and non-fearful response may be more likely to represent an appropriate reaction to potentially unpleasant, yet otherwise benign bodily sensations. Nevertheless, the effects of different demonstrator’s responses to interoceptive stimuli have not yet been systematically examined.

As outlined previously, it is conceivable that observational learning experiences may also differentially contribute to the development of pathological fear and anxiety. Since Rachman [8], etiological theories of anxiety disorders have recognized observational learning as a mechanism in the development of pathological fear and anxiety, suggesting observational learning to modulate the risk for the development of pathological fear and anxiety [9]. Specifically, in the context of describing the etiology of panic disorder, Bouton, Mineka, and Barlow [10] propose that observational learning involving fearful responses to bodily sensations, especially during childhood, may increase the risk for the development of the disorder by sensitizing the observers, i.e., facilitating subsequent fear learning. One possible explanation is that observational learning increases the perceived threat or potential danger of actually benign bodily sensations. Alternatively, observational learning may directly contribute to the acquisition of excitatory fear associations in the observers, which may be easily retrieved when they are later exposed to the same stimuli. While the authors assume that these mechanisms are not mutually exclusive, they propose that both mechanisms can result in sensitization of the observers to subsequent fear acquisition. While observational fear learning is thought to facilitate subsequent fear learning, positive modelling is assumed to potentially counteract fear learning in the future [7]. While systematic empirical evidence for the underlying mechanisms is missing, it is assumed that positive modelling can result in the acquisition of fear inhibiting associations. When retrieved during later fear evoking situations, these inhibitory associations are assumed to potentially counteract later fear acquisition [7], [11]. When such learning experiences take place before an individual is exposed to a novel stimulus, the inhibition of future fear learning has also been referred to as immunization [6], [7]. However, as explained before, the effect of a rather neutral, not mutually positive response, remains to be determined. In sum, learning theories propose that prior observational learning experiences can interact with subsequent direct learning experiences, thereby shaping the risk for the development of pathological fear and anxiety by either facilitating or counteracting fear learning in the future.

Basic research findings lend preliminary support for the modulation of direct fear learning depending on the type of prior observational learning experience, i.e., whether a demonstrator responds calm or fearfully to a novel stimulus. On the one hand, Mineka and Cook [12] revealed that observational learning from a conspecific interacting fearfully with a snake facilitated subsequent observational fear learning to the same stimulus relative to monkeys that were previously exposed to a calm, non-fearful conspecific. As for human studies, initial evidence for the sensitization through observational learning has emerged from retrospective questionnaires studies, revealing that individuals with panic disorder reported a significantly higher frequency of observational learning experiences involving fearful reactions to bodily sensations in youth relative to healthy controls [13]. On the other hand, Mineka and Cook [12] also found that observational learning can counteract the acquisition of fear in rhesus monkeys. Following observation of a conspecific interacting non-fearfully with snakes, the observers acquired less or even no fear when they later watched another monkey interacting fearfully with the snake. In a human study, Golkar and Olsson [6] used video material featuring a demonstrator watching unreinforced presentations of two conditioned stimuli (CSs). The authors found that observing a demonstrator going through unreinforced presentations of two conditioned stimuli (CSs) prevented subsequent observational fear learning in the observer as indicated by the absence of CS + /CS - differences in SCRs, which was interpreted as an immunization effect. While these studies suggest a modulating role of observational learning on subsequent observational fear learning, the effects of observing different demonstrators’ responses to the same interoceptive experience and how they interact with later *direct* fear acquisition have not yet been put to test empirically. Therefore, we aimed to provide an empirical evaluation of different demonstrator responses to temporary dyspnea as an experimental analogue to study different observational learning experiences and how they shape future interoceptive fear conditioning.

For this goal to be met, the protocol by Alcan et al. [4] was extended to study two different observational learning conditions, i.e., a calm, non-fearful vs. a fearful demonstrator response. We used a between-group design in which two observational learning conditions were contrasted to a control condition in which participants saw the screen on which the observational CSs were presented without presence of a demonstrator [comp. 6]. Afterwards, all participants completed a direct acquisition and extinction protocol, with the same CSs that were presented to the demonstrator during observational learning and first-hand temporary dyspnea as the US. Direct fear acquisition and extinction followed an established protocol, that was successfully developed to examine interoceptive fear learning in a laboratory setting [14]. Two geometrical shapes served as CS-stimuli (CS + vs. CS -) and inspiratory resistive loads (IRLs) were used to induce temporary dyspnea (US). We expected that observers of both, the calm and fearful demonstrator group would learn the stimulus contingency, i.e., the association between one geometrical shape and the occurrence of temporary dyspnea for the demonstrator (H1). We assumed that this would be reflected in greater US-expectancy and anxiety ratings for CS + relative to CS - at the end of the observational learning phase and at the beginning of direct acquisition. We also expected differential fear responses to manifest in physiological indicators of fear at the end of observational learning and the beginning of direct acquisition. Specifically, we expected a greater startle eyeblink magnitude, indexing increased defensive mobilization, as well as greater autonomic arousal for CS + relative to CS -, measured by SCRs. For participants of the control condition, we expected no differences in fear responses between CS + and CS - in the indicated points and parameters, because no information about the stimulus contingencies with any kind of emotional relevance was available in the absence of a demonstrator. In line with the literature on interoceptive fear learning (see [5] for a review on interoceptive fear conditioning), we assumed that participants of all conditions would acquire a differential fear responses as indicated by greater anxiety and US-expectancy ratings, greater startle eyeblink magnitudes, and SCRs for CS + relative to CS - that would show at the end of direct acquisition and at the beginning of direct extinction (H2). At the end of extinction, we expected to see no more CS + /CS - differences in verbal reports or physiological measures of fear (H3). In addition to the primary physiological outcome measures, i.e., eyeblink startle response magnitudes and SCRs, literature suggests that direct fear learning can also manifest in cardiac [e.g., 15] and respiratory measures [e.g., [16], [17]], which is why we analyzed and reported these measures in a complementary manner. However, we note that we were not aware of any evidence regarding the effects of observational learning on cardiac and respiratory responses, which is why these outcome measures are analyzed both in an exploratory manner.

Although the existing literature implies that direct fear learning may be differentially modulated by the demonstrator’s response in a preceding observational learning phase, we had no directed hypotheses about the effects of a calm demonstrator's response to an aversive interoceptive sensations, i.e., temporary dyspnea, and aimed to explore the effects of observing a calm relative to a fearful demonstrator’s response to temporary dyspnea on subsequent direct fear learning. Accordingly, throughout all experimental phases, differences in response patterns between the two observational learning groups were examined in an exploratory manner.

## 2 Method

### 2.1 Participants

An a priori power analysis was conducted with G*Power (v.3.11) [18] assuming a small to medium effect size (*f*= 0.2) and defining a power of.80 (1 - β) and an α level probability of.05. The analysis indicated a required total sample size of at least 66 participants. To correct for potential attritions in the sample size during the experiment due to technical problems or excessive recording artifacts, we aimed to assess 75 participants.

The final sample was recruited between 2022 and 2023 and involved *N* = 74 participants of which *n* = 43 self-identified as female and *n* = 31 as male. The mean age was 24.4 years (*SD* = 4.77, range = 18–39). Based on the investigator’s observation, all participants were Caucasian people. Note that ethnic or racial self-identification, as well as nativity are not routinely collected in Germany, and no such data was obtained in the present study. All participants had obtained a general higher education entrance qualification, and 43.3% held a university degree or completed professional training. Most participants (86.5%) were students or in training, while 13.5% were employed or self-employed persons. Participation was remunerated with course credit or financial compensation (15 EUR). The experimental groups did not differ in age, gender distribution or any relevant self-report measure (see Table S1). All inclusion and exclusion criteria are reported in the supplement.

### 2.2 Apparatus

Bioamplifiers (Biopac Systems, Inc., Goleta, California, USA) registered electromyographic activity over the left musculus orbicularis oculi, electrodermal and electrocardiographic activity. A modified ZAN 600 system (nSpire Health, Inc., Oberthulba, Germany) recorded respiratory measures as described in the supplement. Data acquisition and stimulus presentation was controlled with Presentation® software (Version 23.0, Neurobehavioral Systems, Inc., Berkeley, CA, www.neurobs.com).

### 2.3 Stimulus material and procedure

**Fig. 1:**
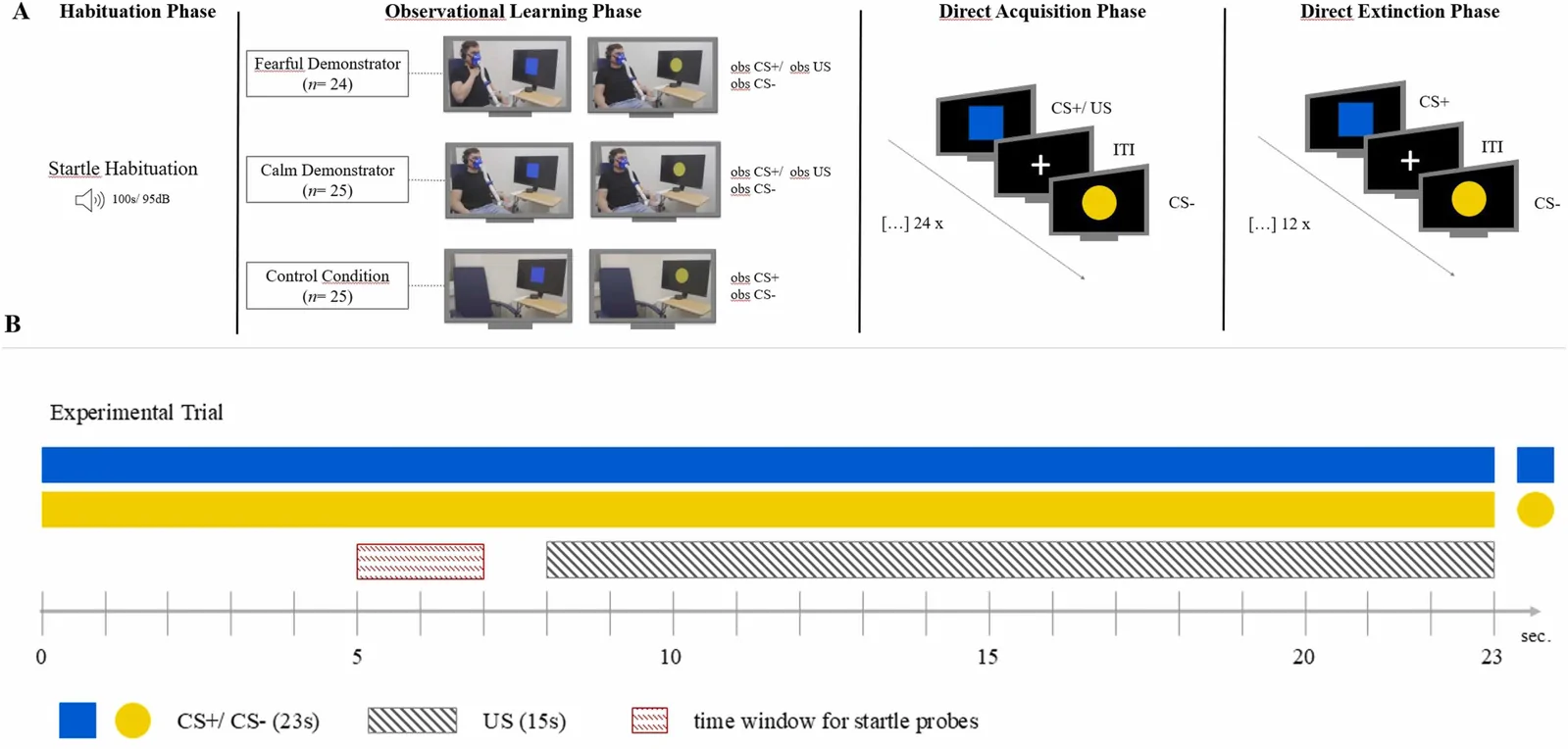
Overview of the design and experimental phases. Panel A depicts the 4 different experimental phases and experimental groups. Before observational learning, all participants completed a 100 s startle habituation phase. Observational learning and direct acquisition comprised 12 CS + and 12 CS - trials, extinction 6 CS + and CS - trials. Participants of all groups saw both CSs presented on the screen for in the experimental room (observational learning) or directly presented them on the screen (acquisition and extinction). Panel B depicts the timing within each trial. Each trial lasted for 21 s during observational learning, and for 23 s during acquisition and extinction.

The experiment comprised four experimental phases (see Fig. 1, Panel A). To examine the influence of different demonstrator responses to temporary dyspnea we created standardized video material featuring a demonstrator completing differential interoceptive fear conditioning in the same experimental room, in which the experiment was conducted. Participants were randomly assigned to either observe a calm, non-fearful vs. a fearful demonstrator’s response to temporary dyspnea, or no demonstrator (control condition). For observers of the calm and fearful demonstrator group, observational CS + trials were partially reinforced with temporary dyspnea for the demonstrator, resulting in two different video sequences for the observational learning phase. For the fearful demonstrator group, the video sequence selected for reinforced observational CS + trials depicted the demonstrator reacting with an anxious facial expression while nervously moving his hand towards his chest and throat. For the calm demonstrator group, the corresponding video sequence depicted the demonstrator reacting with a transient change in facial expression indicating that he had recognized the induced dyspnea but then returning to a neutral facial expression not showing any gross body movements while continuing to breathe in and out regularly. For reinforced CS + trials, the IRL (US) was applied to the demonstrator 8 s after CS-onset and the demonstrator showed noticeably heavier breathing for 13 s, which varied depending on the experimental group as described previously. For unreinforced observational CS + , the same video sequence was used for both observational learning groups, depicting the demonstrator during regular, unrestricted breathing throughout the presentation of the CS + . The same video sequence was also used in both experimental groups for observational CS - trials, again, depicting the demonstrator during regular, unrestricted breathing during the presentation of the CS-. In order to control prior experiences with the CS-stimuli, we also filmed a control condition, showing an empty seat, i.e., no demonstrator, in front of the screen on which CS + and CS - were presented. See also the supplement for additional information about the video material. Two neutral geometrical shapes served as CS-stimuli (blue square, yellow circle). During all experimental phases, trials were separated by ITIs (23 – 27 s), during which a white fixation cross was presented on a black background. During observational learning, the CSs were presented on the screen for the demonstrator and 9 out 12 CS + trials were paired with temporary dyspnea for the demonstrator (75% reinforcement). Timing can be inferred from Fig. 1, Panel B.

After observational learning, participants rated the perceived intensity and unpleasantness of temporary dyspnea for the demonstrator on a 6-point Likert scale (1 *“not at all”* – 6 *“maximum bearable”),* the experienced arousal during the presentation of the CSs on a 9-point Likert-scale (1 *“calm”* – 9 *“excited”*), the experienced anxiety or discomfort during the presentation of CSs on a 6-point Likert-scale (1 *“no anxiety or discomfort at all”* – 6 *“extreme anxiety or discomfort”*), as well as the US-expectancy for the demonstrator (*0% - 100%*). Then, participants completed direct fear acquisition and extinction, during which CSs were directly presented on the screen for 23 s. Before each trial started, participants saw a miniature picture of the geometrical shape to be presented in the upcoming trial and rated the probability (*0% - 100%),* and how anxious they feel to experience temporary dyspnea during the next trial on a 6-point Likert-scale (1 *“no anxiety at all”* – 6 *“extreme anxiety”*), followed by the presentation of the fixation cross for 3 s. During acquisition, 9 out of 12 CS + trials were paired with 15 s of moderate dyspnea (unconditioned stimulus, US), induced via a nylon flow resistor (160 cmH₂O/l/s) connected to the inspiratory port of the breathing circuit (see the supplement for details). During extinction, CS + trials were no longer paired with temporary dyspnea. At the end of the experiment, observers of the calm and fearful demonstrator rated the credibility of the demonstrator’s response during observational learning on a 6-point Likert-scale (1 “*not at all credible”* – 6 *“very credible”*). Further details about instructions and procedures are reported in the supplement.

A 50 ms burst of white noise (95 dB, rise/ fall time < 1 s) was presented binaurally via headphones (AKG-K72 headphones) to elicit a startle response. Six startle probes were delivered during habituation. During observational learning and direct acquisition, 9 startle probes were delivered during each CS and during ITIs, and 4 startle probes for each CS and during ITIs during extinction (5–7 s after CS-onset, 17–21 s after ITI-onset).

### 2.4 Data reduction and analyses

The EMG signal was filtered, rectified, and smoothed offline. The onset (20–100 ms after probe delivery) and peak amplitude (within 150 ms after probe delivery) of the signal were manually determined. Within each participant, raw startle response magnitudes (in µV) were T-standardized (T = 50 +10*z) (see [19]), and averaged across blocks of three trials for observational learning and direct acquisition and across blocks of two trials for extinction.

SCRs were manually determined by the first-interval anticipatory response (FAR), with an onset occurring between 0.9 and 4 s, and peaking between 0.9 and 5.5 s [20]. SCRs were log-transformed, range-corrected, and averaged across blocks of four trials for observational learning and acquisition and across blocks of three trials during extinction.

Further information about physiological recordings and data reduction, including electrocardiogram and respiratory measures, are reported in the supplement.

All analyses were performed in RStudio for Windows [21] (Version 2023.06.1) using *tidyverse* [22]. The R-packages *lme4* [23], *lmerTest* [24], and *emmeans* [25] were used for analyses. To test the pre-registered hypotheses, linear mixed models were fitted for each dependent variable and experimental phase using Restricted Maximum Likelihood Estimation. Group (control condition vs. calm demonstrator vs. fearful demonstrator) and stimulus-type (CS- vs. CS+ for SCR and subjective ratings, ITI vs. CS- vs. CS+ for startle response magnitudes) were coded as factors and were entered to the fixed part of the model. For the direct acquisition and extinction phase, experimental block was coded as factor and also entered as a fixed effect. All two- and three-way-interactions were added to the fixed part of the models. Note that subjective ratings for observational learning were only assessed at the end of the phase. In the random part of the models, intercepts were allowed to vary between participants. Significant increases in model fit following the introduction of random slopes were evaluated using Likelihood-Ratio tests. If the introduction of random slopes significantly increased the model fit (*p* < .05), the more complex model was retained. All fixed effects were evaluated using ANOVA F-tests as implemented in *lme4*. Significant interactions were hierarchically decomposed by analyzing estimated marginal means (simple effects) using *emmeans* [25]. If applicable, we applied Tukey or Sidak adjustments for post-hoc tests as implemented in *emmeans*. For all analyses, a significance level of *p* < .05 was obtained.

In addition to testing confirmatory hypotheses, we conducted planned exploratory analyses as described in the pre-registration in order to compare differences between the two observational learning groups. To this end, we fitted additional linear mixed models that included only the two observational learning groups, adopting the same approach as described above. Results from the exploratory analyses are reported at the end of the respective paragraphs when they differed from the results of the confirmatory analyses.

### 2.5 Transparency and openness

We report how we determined our sample size, all data exclusions, manipulations and measures in the study. The study protocol was pre-registered on the Open Science Framework (OSF, https://osf.io/gkm9f). Analysis code and de-identified data are hosted on OSF (https://osf.io/aqnmf/). Stimulus material used for observational learning can be made available on request. Supplemental material for this article is available online. The study protocol was approved by the ethics committee of the Philipps University Marburg (2022–17k) and was carried out in accordance with the Declaration of Helsinki [11]. The privacy rights of human subjects have been observed at all times.

## 3 Results

Overall, we found a comparable pattern of results for US-expectancy and anxiety ratings, which is why analyses for anxiety ratings are reported in the supplement. For the main outcome measures, the results for confirmatory and exploratory analyses are summarized in Table 1 and Table 2, respectively. For a summary of complementary analyses, see Table S3 in the supplement.

**Table 1:** Overview of the Statistical Significance for Confirmatory Hypotheses Tests.

Experimental Phase	Parameter	*F* Test	*F(df)*	*p*	*η*_*p*_^*2*^
Observational Learning Phase					
	US-Exp.	Stimulus x Group	7.14 (1, 94)	.009 **	.07
	Anxiety	Stimulus x Group	12.64 (2, 71)	< .001***	.26
	SCR	Stimulus x Group Block x Stimulus x Group	0.98 (2, 276.0) 0.61 (4, 276.0)	.377 .659	< .01 < .01
	Startle Eyeblink	Stimulus x Group Block x Stimulus x Group	9.04 (4, 460.8) 0.69 (8, 460.5)	< .001*** .797	.07 < .01
Direct Acquisition Phase					
	US-Exp.	Stimulus x Group Block x Stimulus x Group	13.19 (2,71.0) 7.07 (4, 213.0)	< .001*** < .001***	.27 .12
	Anxiety	Stimulus x Group Block x Stimulus x Group	5.37 (2, 71) 1.60 (4, 284.0)	.007** .175	.13 .02
	SCR	Stimulus Stimulus x Group Block x Stimulus x Group	30.71 (1, 91.7) 0.05 (2, 91.7) 1.35 (4, 207.0)	< .001*** .951 .254	.25 < .01 .03
	Startle Eyeblink	Stimulus Stimulus x Group Block x Stimulus x Group	56.5 (1, 65.0) 1.46 (4, 65.1) 0.84 (8, 317.2)	< .001*** .226 .571	.63 .08 .02
Direct Extinction Phase					
	US-Exp.	Stimulus x Group Block x Stimulus x Group	2.38 (2,71.0) 3.91 (2, 70.9)	.100 .024*	.06 .10
	Anxiety	Stimulus x Group Block x Stimulus x Group	3.12 (2,71.0) 6.16 (2, 71.0)	.051 .003**	.08 .15
	SCR	Stimulus Stimulus x Group Block x Stimulus x Group	0.78 (1,1 29.6) 0.22 (2, 129.6) 0.02 (2, 207.0)	.380 .806 .985	< .01 < .01 < .01
	Startle Eyeblink	Block x Stimulus Stimulus x Group Block x Stimulus x Group	3.53 (2, 310.3) 0.79 (4, 310.3) 0.39 (4, 310.3)	.031* .546 .819	.02 < .01 < .01

*Note.* Block was not included for subjective ratings in the first experimental phase. US-Exp.: US-expectancy ratings (not applicable for the control group during the first phase), Anxiety: anxiety ratings; SCR: skin conductance response; Startle Eyeblink: eyeblink startle response magnitudes. *p < .05. **p < .01. ***p < .001.

**Table 2:** Overview of the Statistical Significance for Exploratory Analyses.

Experimental Phase	Parameter	*F* Test	*F(df)*	*p*	*η*_*p*_^*2*^
Observational Learning Phase					
	US-Exp.	Stimulus x Group	7.14 (1,94.0)	.009 **	.07
	Anxiety	Stimulus x Group	3.00 (1,47.0)	.089	.06
	SCR	Block x Group Stimulus x Group Block x Stimulus x Group	3.88 (2230.0) 0.51 (1230.0) 0.13 (2230.0)	.022* .476 .877	.05 < .01 < .01
	Startle Eyeblink	Block Stimulus Stimulus x Group Block x Stimulus x Group	9.04 (2,61.5) 0.69 (2305.3) 1.17 (2305.3) 0.44 (4304.8)	< .001*** < .001*** .313 .779	.40 .13 < .01 < .01
Direct Acquisition Phase					
	US-Exp.	Stimulus x Group Block x Stimulus x Group	1.54 (1,47.0) 3.91 (2141.0)	.221 .022*	.03 .05
	Anxiety	Block Stimulus Stimulus x Group Block x Stimulus x Group	11.96 (2235.0) 245.50 (1235.0) 0.60 (1235.0) 0.53 (2235.0)	.001*** < .001*** .440 .591	.09 .52 < .01 < .01
	SCR	Block x Stimulus Stimulus x Group Block x Stimulus x Group	3.65 (2138.0) 0.07 (1,50.7) 0.03 (2138.0)	< .001*** .792 .974	.05 < .01 < .01
	Startle Eyeblink	Block Stimulus x Group Block x Stimulus x Group	26.36 (2,44.0) 3.25 (2,45.3) 0.85 (4217.7)	< .001*** .048* .495	.55 .13 .02
Direct Extinction Phase					
	US-Exp.	Block x Stimulus Stimulus x Group Block x Stimulus x Group	88.0 (1,94.0) 0.21 (1,47.0) 0.73 (1,94.0)	< .001*** .647 .397	.48 < .01 < .01
	Anxiety	Block x Stimulus Stimulus x Group Block x Stimulus x Group	23.3 (1,47.0) 0.70 (1,47.0) 0.41 (1,47.0)	< .001*** .408 .525	.33 .01 < .01
	SCR	Stimulus x Group Block x Stimulus x Group	0.46 (1,74.6) 0.01 (1,92.0)	.502 .939	< .01 < .01
	Startle Eyeblink	Block Stimulus Stimulus x Group Block x Stimulus x Group	11.36 (1219.3) 42.75 (2, 218.5) 0.48 (2218.5) 0.58(2, 218.5)	< .001*** < .001*** .619 .558	.05 .28 < .01 < .01

*Note.* Summary of results for statistical models including only observers of the calm vs. fearful demonstrator. Block was not included for subjective ratings in the first experimental phase. US-Exp.: US-expectancy ratings (not applicable for the control group during the first phase), Anxiety: anxiety ratings; SCR: skin conductance response; Startle Eyeblink: eyeblink startle response magnitudes *p < .05. **p < .01. ***p < .001.

### 3.1 Manipulation check

There was no significant difference in the perceived credibility of the demonstrator’s response between observers of the fearful, *M*_Fe__arful_= 4.08, *SD*_Fe__arful_= 1.25, vs. calm demonstrator, *M*_C__alm_= 4.32, *SD*_C__alm_= 1.38, *t*(47.0)_*Fearful>Calm*_= 0.63, *p* = .531, *d*= 0.18, 95%CI [−0.37, 0.79] and groups did not differ in the overall perception of respiratory symptoms in the demonstrator (see Supplement B). Credibility ratings indicated that participants generally perceived the demonstrator’s response as rather credible, with ratings centered in the upper half of the possible score range, *MD* = 4.00, *IQR* = 2.00, *Q1* = 3.00, *Q3* = 5.00.

### 3.2 Arousal ratings after observational learning

At the end of the observational learning phase, the calm and fearful demonstrator group reported greater arousal to CS + vs. CS- (see supplementary Table S1), with no differences between both groups, all *t*s < 1.80, all *p*s > .174 Observers of the fearful but not calm demonstrator showed higher CS + /CS- differences in reported arousal as compared to observers of the control condition, *t*(142)_*Fearful>Control*_= 3.63, *p* = .001. Observers of the fearful demonstrator reported greater arousal in response to CS + than individuals in the control condition, *t*(142)_*Fearful>Control*_= 2.78, *p* = .017, while reported arousal did not differ in response to CS-, *t*(142)_*Fearful>Control*_= −2.35, *p* = .052, *F*(2142)_*StimulusxGroup*_= 6.60, *p* = .002, *η*_*p*_^*2*^= .09, 95%CI [0.02, 1.00]. Results for reported valence largely mirrored the pattern previously reported for arousal ratings and are reported in supplement B.

### 3.3 Observational learning

#### 3.3.1 Ratings of demonstrator’s response

**Fig. 2:**
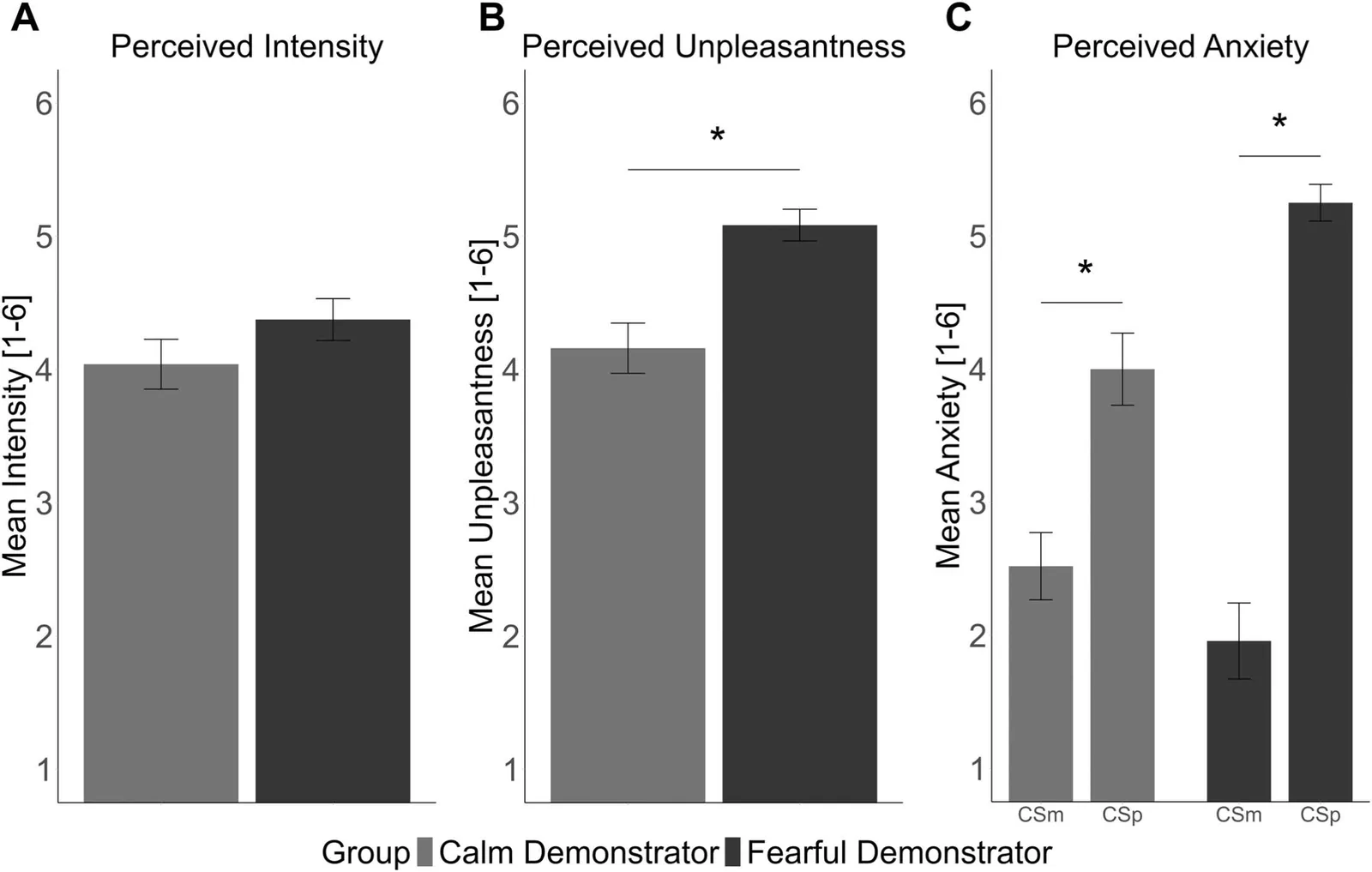
Perception of the demonstrator’s response assessed at the end of the observational learning phase. CS= conditioned stimulus. Error bars represent the standard error of the mean. Note that ratings were not applicable for the control condition (no demonstrator).

After observational learning, there was no difference in the perceived intensity of dyspnea between observers of the calm and fearful demonstrator, *t*(46.0)_*Fearful>Calm*_= 1.37, *p* = .177, *d*= 0.39, 95%CI [−0.16,1.04]. However, dyspnea was perceived as more unpleasant by observers of the fearful vs. calm demonstrator, *t*(40.3)_*Fearful>Calm*_= 4.14, *p* < .001, *d*= 1.85, 95%CI [0.68,1.85]. Moreover, observers of the fearful vs. calm demonstrator perceived more anxiety or discomfort in the demonstrator during presentation of the observational CS + , *t*(94.0)_*Fearful>Calm*_= 3.61, *p* < .001, while there was no difference between groups for the observational CS -, *t*(94.0)_*Fearful>Calm*_= −1.62, *p* = .108, *F*(1,94)_*StimulusxGroup*_= 13.72, *p* < .001, *η*_*p*_^*2*^= .13, 95%CI [0.04,1.00]. Ratings of the demonstrator’s response are depicted in Fig. 2.

#### 3.3.2 US-expectancy

**Fig. 3:**
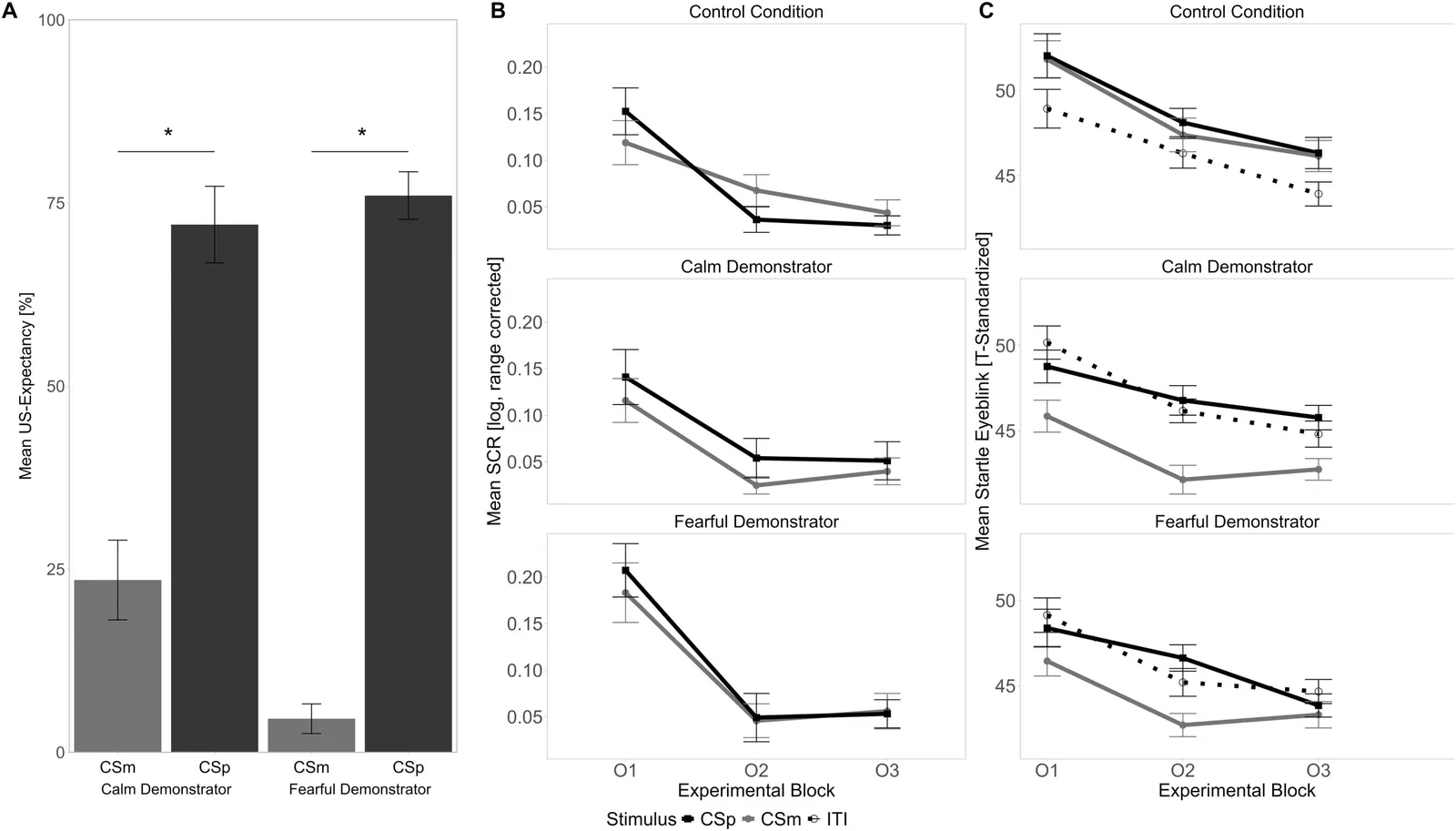
Subjective and psychophysiological responses during observational learning. CS= conditioned stimulus, ITI= inter-trial-interval. O1-O3 = blocks of trials during observational learning, averaged across three trials for startle responses and across blocks of four trials for SCRs. US-expectancy was only assessed once at the end of observational learning and was not applicable for the control condition. Mean responses are depicted for each experimental group, with error bars indicating the standard error of the mean.

At the end of observational learning, participants of both observational learning groups reported greater US-expectancy in response to observational CS + vs. CS-, all *ts*_*CS+>CS-*_> 8.09, all *p*s < .001. However, CS + /CS- differences in US-expectancy ratings were greater for observers of the fearful vs. calm demonstrator, *F*(1,94)_*StimulusxGroup*_= 7.14, *p* = .009, *η*_*p*_^*2*^= .07, 95%CI [0.01,1.00]. While groups did not differ in US-expectancy ratings in response to observational CS + , *t*(94)_*Fearful>Calm*_= 0.65, *p* = .515, observers of the fearful demonstrator reported lower US-expectancy in response to observational CS- as compared to observers of the calm demonstrator, *t*(94)_*Fearful>Calm*_= −3.13, *p* = .002 (see Fig. 3, Panel A).

#### 3.3.3 Skin conductance response

The model for SCRs revealed an overall decline in SCRs during observational learning, *F*(2111.7)_*Block*_= 68.96, *p* < .001, *η*_*p*_^*2*^= .55, 95%CI [0.45, 1.00], that did not differ between stimuli or groups, all *by-stimulus* and *by-group* interactions, all *F*s < 2.37, all *p*s > .057 (see Fig. 3, Panel B).

The exploratory model revealed significantly greater SCRs only during the first block of the observational learning phase for observers of the fearful demonstrator, relative to observers of the calm demonstrator, *t*(85.1)_*Fearful>Calm*_= 2.51, *p* = .014, *F*(2230)_*BlockxGroup*_= 3.88, *p* = .022, *η*_*p*_^*2*^= .03, 95%CI [0.00,1.00].

#### 3.3.4 Startle response magnitudes

During the observational learning phase, participants of the control condition exhibited a significant potentiation of startle response magnitudes for both CSs relative to ITI, all *t*s_*CS+,CS->ITI*_> 3.42, all *p*s < .002, while startle response magnitudes did not differ in response to CS + as compared to CS -, *t*(461)_*CS+>CS-*_= 0.04, *p* = .999. In contrast, we found that startle response magnitudes were greater in response to CS + relative to CS - for observers of the calm and fearful demonstrator, all *t*s_*CS+>CS-*_> 3.06, all *p*s < .007, *F*(4460.8)_*StimulusxGroup*_= 9.04, *p* < .001, *η*_*p*_^*2*^= .07, 95%CI [0.03, 1.00]. Although there was no significant potentiation of startle response magnitudes in response to CS + vs. ITI for observers of the calm and fearful demonstrator, all *t*s_*CS+>ITI*_ < 0.28, all *p*s > .960, both observers exhibited a significant attenuation of startle response magnitudes in response to CS - relative to ITI, all *t*s_*CS->ITI*_> 2.75, all *p*s < .002 (see Fig. 3, Panel C). Startle response magnitudes in response to CS + and CS - did not differ between observers of the calm and fearful demonstrator, all *t*s_*Fearful>Calm*_< 0.69, all *p*s > .767. There was an overall decrease in startle response magnitudes over the course of the observational learning phase, *F*(2105.1)_*Block*_= 30.07, *p* < .001, *η*_*p*_^*2*^= .36, 95%CI [0.24, 1.00], that did not differ between stimuli or groups, all *by-block* interactions, all *F*s < 0.85, all *p*s > .493. The three-way interaction did not reach significance in the model *F*(8460.5)_*BlockxStimulusxGroup*_= 0.58, *p* = .797.

#### 3.3.5 Complementary analyses

During the observational learning phase, no systematic CS + /CS - differences emerged in cardiac responding.

Changes in respiratory rate did not differentiate between CS + and CS - for participants of the control condition, while significant CS + /CS - differences emerged throughout the observational learning phase for observers of any demonstrator. Specifically, at the end of the observational learning phase, observers of the fearful demonstrator exhibited a higher respiratory rate in response to CS + relative to CS -, while observers of the calm demonstrator exhibited an opposite pattern, i.e., higher respiratory rate in response to CS - relative to CS + . We found no systematic changes in tidal volume or minute ventilation in response to CS + relative to CS - during observational learning. All statistical analyses are comprehensively reported in the supplement.

### 3.4 Acquisition

#### 3.4.1 US-expectancy

**Fig. 4:**
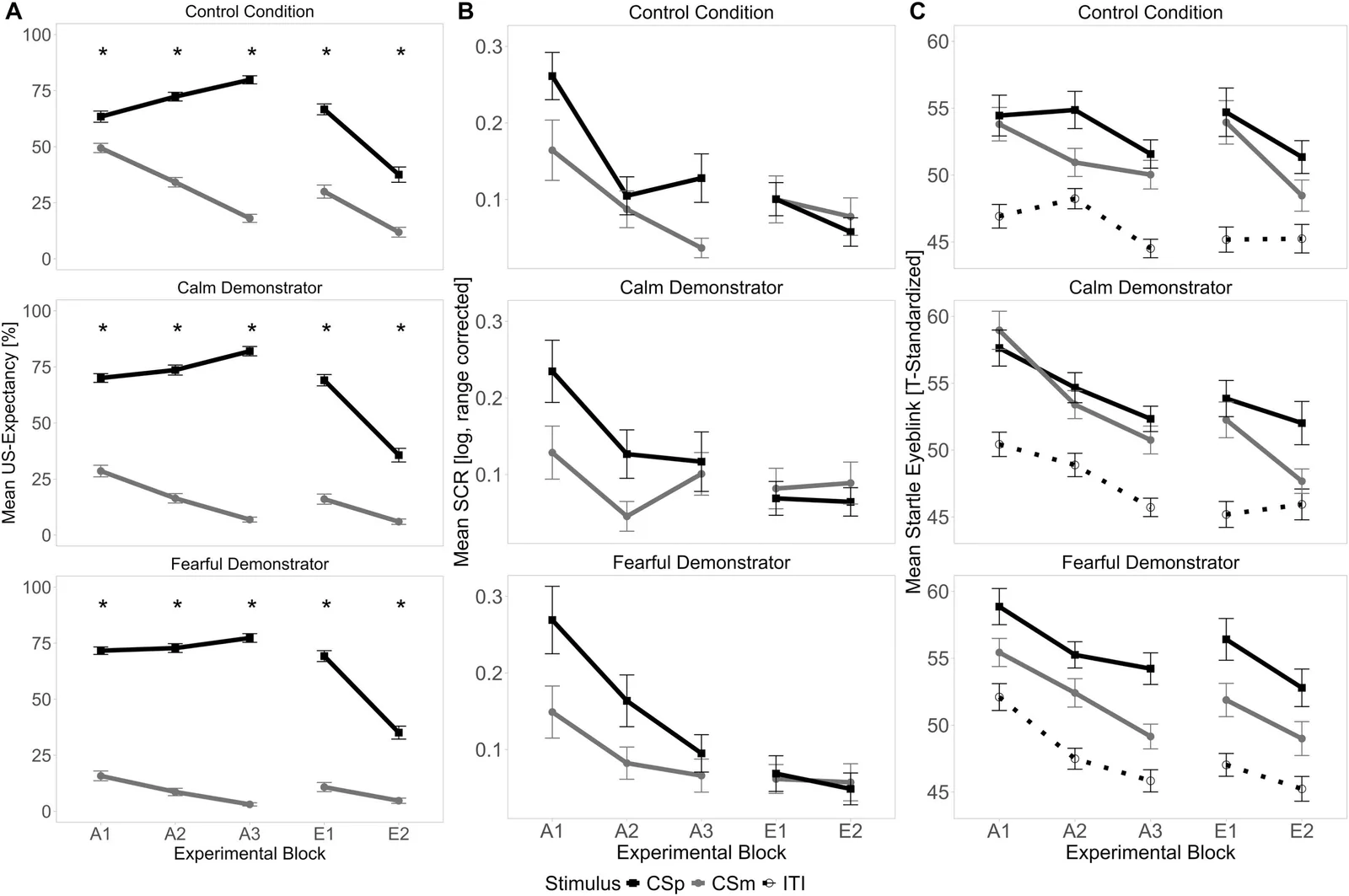
Subjective and psychophysiological responses during acquisition and extinction. CS= conditioned stimulus, ITI= inter-trial-interval. A1-A3 = blocks of trials during acquisition, averaged across three trials for startle responses and across blocks of four trials for SCRs and US-expectancy. E1-E2 = blocks of trials during extinction, averaged across two trials for startle responses and across blocks of three trials for SCRs and US-expectancy. Mean responses are depicted for each experimental group, with error bars indicating the standard error of the mean.

As compared to participants of the control condition, observers of the calm and fearful demonstrator started out with greater CS + /CS - differences at the beginning of the direct acquisition phase, which were also evident during the second block of trials, all *t*s_*Fearful,Calm>Control*_> 3.05, all *p*s < .009. The magnitude of CS + /CS - differences converged for participants of the control condition during the third block of the direct acquisition phase, all *t*s_*Fearful,Calm>Control*_< 2.13, all *p*s < .101*, F*(4213.0)_*BlockxStimulusxGroup*_= 7.07, *p* < .001, *η*_*p*_^*2*^= .12, 95%CI [0.05, 1.00] (see Fig. 4, Panel A). As compared to participants of the control condition, observers of the calm and fearful demonstrator reported smaller US-expectancy in response to CS- during the first and second block of trials, all *t*s_*Fearful,Calm>Control*_> |−3.76|, all *p*s < .001, while US-expectancy did not differ in response to CS + between groups, all *t*s_*Fearful,Calm>Control*_< 1.80, all *p*s > .175. The magnitude of CS + /CS- differences did not differ between observers of the calm and fearful demonstrator during acquisition in the overall model, all *t*s_*Fearful>Calm*_< 2.28, all *p*s < .072.

Following our pre-registered analysis plan specifically comparing responses in observers of the calm and fearful demonstrator, we found greater CS + /CS - differences in the first block of the acquisition phase for observers of the fearful relative to observers of the calm demonstrator, *t*(88.2)_*Fearful>Calm*_= 2.12, *p* = .030, while the magnitude of CS + /CS - differences converged during the second and third block of trials, all *t*s_*Fearful>Calm*_< 1.07, all *p*s > .286, *F*(2188)_*BlockxStimulusxGroup*_= 3.24, *p* = .041, *η*_*p*_^*2*^= .03, 95%CI [0.00, 1.00]. Greater CS + /CS - differences during the first block of trials resulted from smaller US-expectancy in response to CS - for observers of the fearful relative to observers of the control condition, *t*(100.9)_*Fearful>Calm*_= 3.03, *p* = .003, while US-expectancy did not differ in response to CS + , *t*(84.6)_*Fearful>Calm*_= 0.34, *p* = .735.

#### 3.4.2 Skin conductance response

SCRs were greater in response to CS + as compared to CS -, *F*(2,90.8)_*Stimulus*_= 17.53, *p* < .001, *η*_*p*_^*2*^= .28, 95%CI [0.15, 1.00], and equally decreased throughout acquisition, *F*(1,91.7)_*Block*_= 30.70, p < .001, *η*_*p*_^*2*^= .25, 95%CI [0.13, 1.00], *F*(2207.0)_*BlockxStimulus*_= 2.82, *p* = .062, *η*_*p*_^*2*^= .03, 95%CI [0.00, 1.00]. Groups did not differ with regard to the decrease in SCR or CS + /CS - differences, all *by-group* interactions, all *t*s < 1.35, all *p*s > .254, (see Fig. 4, Panel B).

#### 3.4.3 Startle response magnitudes

Throughout the acquisition, we found an overall decline in startle response magnitudes, *F*(2, 73.0)_*Block*_= 27.59, *p* < .001, *η*_*p*_^*2*^= .43, 95%CI [0.29, 1.00] for observers of all groups. Moreover, across observers of all groups, we found a significant potentiation of startle response magnitudes for both CSs relative to ITI, all *t*s_*CS+,CS->ITI*_> 8.25, all *p*s < .001, which was greater in response to CS + relative to CS -, *t*(65.2)_*CS+>CS-*_= 3.00, *p* = .012, *F*(2,65.02)_*Stimulus*_= 56.49,p < .001, *η*_*p*_^*2*^= .63, 95%CI [0.51, 1.00]. Differences in startle response magnitudes did not differ between groups *F*(4,65.8)_*StimulusxGroup*_= 1.46, *p* = .226 and did not change throughout the direct acquisition phase, *F*(4317.2)_*BlockxStimulus*_= 1.56, *p* = .200 (see Fig. 4, Panel C). The three-way interaction did not reach significance, *F*(8317.2)_*BlockxStimulusxGroup*_ = 0.84, *p* = .571.

As compared to the overall model, the model comprising only observers of the calm and fearful demonstrator group revealed significant differences in the startle response magnitudes between groups, *F*(2,45.3)_*StimulusxGroup*_= 3.25, *p* = .048, *η*_*p*_^*2*^= .13, 95%CI [0.00, 1.00]. While participants of both groups exhibited a significant potentiation of startle response magnitudes in response to both CSs relative to ITI, all *t*s_*CS+,CS->ITI*_> 3.89, all *p*s < .001, startle response magnitudes were significantly greater in response to CS + as compared to CS - for observers of the fearful demonstrator, *t*(45.2)_*CS+>CS-*_= 3.52, *p* = .003, while no CS + /CS - difference in startle response magnitudes occurred for observers of the calm demonstrator, *t*(45.3)_*CS+>CS-*_= 0.25, *p* = .965. No other two- or three-way interaction reached significance in the model, all *F*s < 0.97, all *p*s > .422.

#### 3.4.4 Complementary analyses

**Fig. 5:**
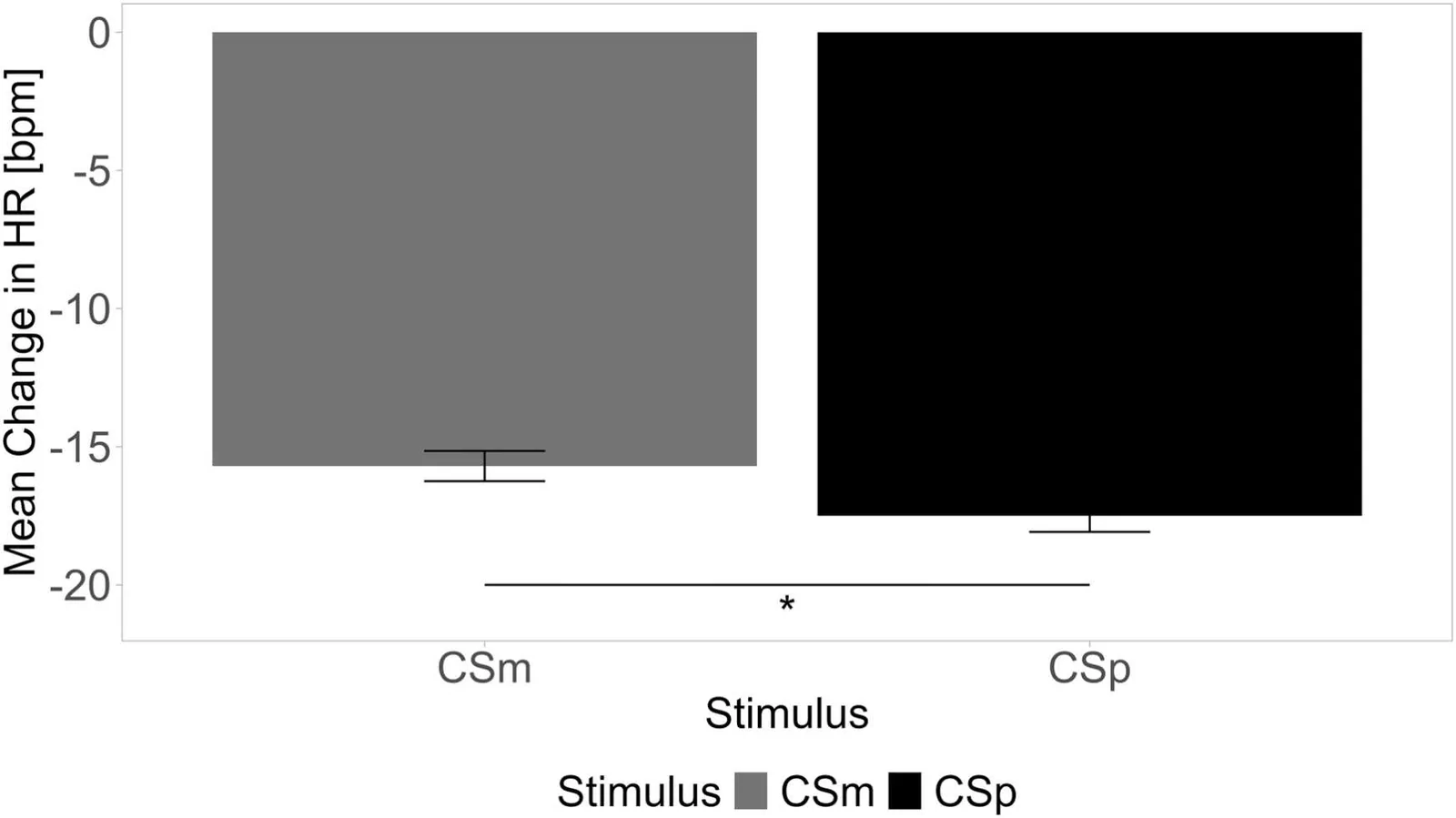
Mean changes in late cardiac deceleration (D2-component) during the direct acquisition phase (bpm). Changes in cardiac deceleration are averaged across groups and all blocks of the direct acquisition phase. Error bars indicate the standard error of the mean.

**Fig. 6:**
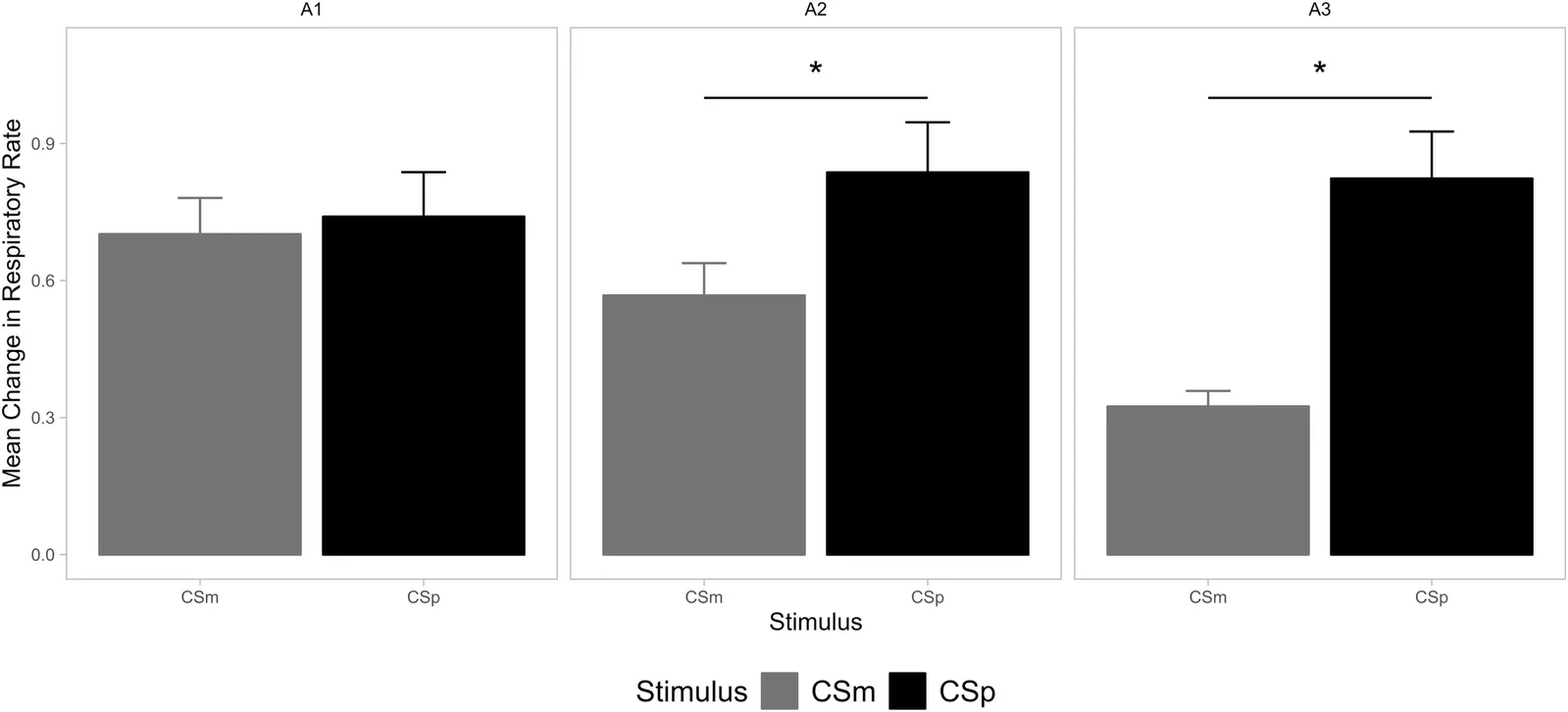
Mean changes in respiratory rate (breaths per minute) for each experimental group averaged across 8 s of CS-presentation and all blocks of trials in the direct acquisition phase. Error bars represent the standard error of the mean.

Throughout the direct acquisition phase, no systematic changes in initial cardiac decelerations and cardiac acceleration in response to CS + relative to CS - emerged for participants of any group. We found that late cardiac deceleration was overall more pronounced in response to CS + relative to CS -, which however, did not change during the direct acquisition phase and did not differ between groups (see Fig. 5). Moreover, we detected an overall increase in respiratory rate during CS-presentation that was relatively more pronounced in response to CS + relative to CS - starting 6 s after CS-onset, and CS + /CS - differences in changes of respiratory rate became more pronounced upon the end of the acquisition phase (see Fig. 6). These patterns did not differ between groups. While we found an overall increase in tidal volume relative to baseline during CS - presentation 6 s after onset, CS + /CS - differences in tidal volume did not emerge systematically throughout the direct acquisition phase for participants of any group. While we found an overall stronger increase in minute ventilation in response to CS + for observers of the fearful demonstrator as compared to observers of the calm demonstrator during the first block of trials, changes in minute ventilation in response to CS + relative to CS - did not evolve systematically or differed between groups during the direct acquisition phase. All statistical analyses are reported in the supplement.

### 3.5 Extinction

#### 3.5.1 US-expectancy

Observers of the fearful demonstrator group exhibited significantly greater CS + /CS - differences in US-expectancy ratings as compared to participants of the control at the beginning of extinction, *t*(104)_*Fearful>Control*_= 3.06, *p* = .009, that converged upon the end of extinction, *t*(104)_*Fearful>Control*_= 0.662, *p* = .882. Greater CS + /CS - differences during the first block of trials for observers of the fearful demonstrator as compared to participants of the control condition resulted from smaller US-expectancy in response to CS -, *t*(89.2)_*Fearful>Control*_= −4.12, *p* < .001, while there was no difference in US-expectancy in response to CS + between the two groups, *t*(86.2)_*Fearful>Control*_= 0.51, *p* = .865. There was no difference in the magnitude of CS + /CS - differences in US-expectancy ratings between observers of the calm and fearful demonstrator throughout extinction, all *t*s_*Fearful>Calm*_< 0.78, all *p*s > .835, and no difference between observers of the calm demonstrator and participants of the control condition, all *ts*_*Fearful>Calm*_< 2.37, all *p*s > 0.064, *F*(2,71.0)_*BlockxStimulusxGroup*_= 3.91, *p* = .025, *η*_*p*_^*2*^= .10, 95%CI [0.01, 1.00] (see Fig. 4, Panel A).

#### 3.5.2 Skin conductance response

SCRs did not decline throughout extinction, *F*(1198.7)_*Block*_= 1.95, *p* = .164, *η*_*p*_^*2*^< .01, 95%CI [0.00, 1.00], and there were no differences between stimuli or groups, all *F*s < 1.95, all *p*s > .164 (see Fig. 4, Panel B).

#### 3.5.3 Startle response magnitudes

At the beginning of extinction, participants of all groups exhibited a significant potentiation of startle response magnitudes to both CSs relative to the ITI, all *t*s_*CS+,CS->ITI*_> 6.35, all *p*s < .001, with no differences in startle response magnitudes between CS + and CS-, *t*(311)_*CS+>CS-*_= 1.65, *p* = .228. Towards the end of extinction, startle response magnitudes significantly decreased in response to both CS + and CS -, all *t*s_*E1>E2*_ > 2.44, *p* < .015, but not in response to ITI, *t*(309) _*E1>E2*_= 0.36 < 1.90, *p=* .7168, *F*(2310.3)_*BlockxStimulus*_= 3.53, *p* = . 030, *η*_*p*_^*2*^= . 02, 95%CI [0.00, 1.00]. At the end of extinction, a significant potentiation of startle responses was maintained in response to both, CS + and CS - relative to ITI, all *t*s_*CS+,CS->ITI*_ > 2.66, all *p*s < .022. Moreover, we found that startle response magnitudes were greater in response to CS + relative to CS - at the end of extinction, *t*(310)_*CS+>CS-*_ = 3.31, *p* = .003. Startle response magnitudes did not differ between groups during extinction, all *by-group interactions*, all *F*s < 0.78, all *p*s > .546 (see Fig. 4, Panel C).

#### 3.5.4 Complementary analyses

During the direct extinction phase, we detected no systematic changes in cardiac responding in response to CS + relative to CS- in any component or group.

While changes in respiratory rate decreased throughout the direct extinction phase, tidal volume and minute ventilation still increased during CS-presentation for participants of all groups. Yet, no systematic changes in respiratory rate, tidal volume and minute ventilation in response to CS + relative to CS- emerged over the course of direct extinction for participants of any group. See also the supplement for the detailed report of all statistical analyses.

## 4 Discussion

The present study sought to systematically evaluate the impact of prior observational learning experiences involving different demonstrator responses to temporary dyspnea on the subsequent acquisition of fear of interoceptive threat Table 3 presents our pre-registered hypotheses and primary outcomes measures, summarizing supported predictions and deviations thereof. Observational learning, relative to the absence of such experiences, was followed by greater US-expectancy and subjective anxiety ratings in response to CS + relative to CS - at the end of the observational learning phase, which was also evident in US-expectancy ratings at the beginning of the direct acquisition phase. Throughout the direct acquisition phase differential fear responses were found for participants of all groups, as indicated by greater US-expectancy, reported anxiety, autonomic arousal and startle response magnitudes in response to CS + relative to CS - and a complementary pattern of results in cardiac and respiratory measures. Notably, additional exploratory analyses revealed that observers of the fearful relative to the calm demonstrator started out with greater CS + /CS - differences in US-expectancy ratings during the direct acquisition phase. Moreover, these analyses also revealed that observers of the fearful demonstrator exhibited significantly greater startle response magnitudes to CS + relative to CS -, while no CS + /CS - differences were found in observers of the calm demonstrator during the direct acquisition phase. During extinction we found a significant decline in fear responses across all of our primary outcome measures except for SCRs. Yet, participants of all groups reported greater US-expectancy and anxiety and maintained greater startle response magnitudes in response to CS + as compared to CS - until the end of the extinction phase.

**Table 3:** Overview of Pre-Registered Hypotheses and Key Findings.

**H1:** At the end of the observational learning phase and at the beginning of the direct acquisition phase, observers of the calm and fearful demonstrator group would show greater fear responses to CS + relative to CS-. No significant CS + /CS- differences were expected for participants of the control condition.				
	US-Exp.	Anxiety	SCR	Startle
Observational Learning Phase	✓	✓	✗	✗
Direct Acquisition Phase	✓	✓	✗	✗

**H2:** At the end of the direct acquisition phase and at the beginning of the direct extinction phase, participants of all groups would show greater fear responses to CS + relative to CS-.				
	US-Exp.	Anxiety	SCR	Startle
Direct Acquisition Phase	✓	✓	✓	✓
Direct Extinction Phase	✓	✓	✗	✗

**H3:** At the end of the direct extinction phase, fear responses would no longer differ between CS + relative to CS- for participants of all groups.				
	US-Exp.	Anxiety	SCR	Startle
Direct Extinction Phase	✗	✗	✓	✗

*Note.* US-Exp.: US-expectancy ratings (not applicable for the control group during the first phase), Anxiety: anxiety ratings; SCR: skin conductance response; Startle: eyeblink startle response magnitudes.

### 4.1 Observational learning phase

In line with the findings from Alcan et al. [4], observational learning of interoceptive threat was followed by differential fear responses, i.e. greater US-expectancy and anxiety ratings in response to CS + relative to CS -. In contrast, no CS + /CS - differences occurred in subjective ratings in the control condition, i.e., in the absence of such learning experiences, which was expected, because these participants saw only the visual cues, but these cues were not paired with an aversive outcome (i.e., temporary dyspnea) during the first phase of the experiment. At the end of the observational learning phase, observers of the fearful demonstrator group exhibited greater CS + /CS - differences in US-expectancy ratings as compared to observers of the calm demonstrator group, which resulted from significantly reduced US-expectancy in response to CS -. We assume that greater CS + /CS - differences in verbal reports likely reflect information about the properties of the US, such as the perceived unpleasantness of temporary dyspnea, that were also conveyed by the different demonstrators’ responses. This notion is in line with Selbing and Olsson [26], proposing that fearful responses of a demonstrator may increase the perceived aversiveness and threat relevance, thereby motivating differential learning. Indeed, we found that the observers’ perception of temporary dyspnea was modulated by the demonstrator’s response, with observers of the fearful demonstrator rating the experience of temporary dyspnea for the demonstrator as more unpleasant as compared to observers of the calm demonstrator. Given that observers of the fearful demonstrator also perceived the demonstrator to be more fearful, this may point towards a higher perceived threat relevance for these observers as compared to [27] observers of the calm demonstrator.

Unexpectedly, during observational learning, we did not find greater startle response magnitudes to CS + as compared to CS - or significant CS + /CS - differences in SCRs and cardiac responding for observers of the calm and fearful demonstrator as compared to participants of the control condition. Although this contradicts our predictions, specifically for SCRs, the present findings align with prior observational learning studies [3], [4], [6], [26], showing that conditioned fear responses in physiological indices were stronger during subsequent direct test stages, and that conditioned CS + /CS - differences first emerged at the beginning of a subsequent direct test phase because of the greater imminence of acute threat. This interpretation is consistent with the findings from other observational learning studies measuring startle response magnitudes [3], [26], in which the absence of potentiation of startle responses in response to CS + was interpreted as indicator for the reduced imminence of threat during observational learning. Still, other studies reported significant CS + /CS - differences in SCRs already during observational learning [1], [28], [29], yet, suggesting that physiological responses during observational learning were also dependent on other factors, such as the observers’ goals, awareness of the CS-US contingency, or the observers’ empathy with the demonstrator. While subjective ratings indicate that observers of the calm and fearful demonstrator group acquired significant contingency knowledge upon the end of the observational learning stage, the influence of the observers’ goals and empathic appraisals on physiological responses remain speculative. Nevertheless, we note that observers were not explicitly instructed that they will subsequently complete the same experiment as the demonstrator, which might have influenced the perceived relevance of learning for the observers [comp. also 4].

Finally, two findings of the observational learning phase are worth mentioning. First, startle response magnitudes in response to CS - were significantly attenuated relative to ITI for observers of both, the calm and fearful demonstrator. Attenuation of startle response magnitudes is typically found for positive stimuli, such as CSs that are paired with appetitive US (e.g., [26]) or stimuli that are signaling the omission of the US (e.g. [28], comp. also [29]). Thus, a potential post-hoc explanation for this observed pattern of results might be that the CS- obtained a rather positive valence, particularly in contrast to CS + , by signaling the absence of temporary dyspnea, likewise for the observers of the calm and fearful demonstrator. Second, at the end of the observational learning phase, observers of the fearful demonstrator exhibited an increase in respiratory rate in response to CS + relative to CS -, which aligns with the findings from previous studies, revealing comparable increases in respiratory rate during the anticipation of threat or during aversive imagery, that were also associated with higher levels of state anxiety, anxiety sensitivity and suffocation fear [16], [27], [30], [31], [32]. Although these changes imply successful differential learning in respiratory measures for observers of the fearful demonstrator, we note that the identified changes were small in magnitude and should be interpreted cautiously. Across participants of all groups, we detected an overall decrease in tidal volume, resulting in a reduction in minute ventilation during CS presentation. This pattern indicates shallower breathing relative to baseline, which however did not change systematically over the course of observational learning. Although we did not observe an overall increase in respiratory rate across groups, descriptive inspection of the respiratory data suggested an opposing pattern of respiratory rate and tidal volume, which may hint at compensatory mechanisms to maintain constant minute ventilation [comp. [16], [27]].

#### 4.1.1 Direct acquisition phase

Consistent with our hypothesis and in line with the study from Alcan et al. [5], observers of both, the calm and fearful demonstrator reported greater US-expectancy to CS + than CS- at the beginning of direct acquisition, indicating that these observers carried over information from observational learning. Although contingency learning also emerged rapidly for participants of the control condition, i.e., greater US-expectancy in response to CS + relative to CS -, the magnitude of CS + /CS - differences converged between groups only at the end of acquisition. In line with US-expectancy ratings at the beginning of the direct acquisition phase, CS + /CS - differences in anxiety ratings were overall greater for observers of the calm and fearful demonstrator as compared to participants of the control condition, although absolute ratings indicated that observers of the fearful demonstrator reported significantly smaller anxiety in response to CS - relative to participants of the control condition and the pattern of anxiety ratings did not change throughout learning. Smaller CS + /CS - differences in US-expectancy ratings for participants of the control condition during the first block of the acquisition phase could be interpreted as indicator of inhibited fear learning as a result of the mere pre-exposure to the CSs stimuli without being paired with an aversive event (i.e., no threat associations could be acquired for any cue) during the first phase of the experiment (latent inhibition, [comp. 33]). Moreover, based on the findings from Lindström, Haaker, and Olsson [30], suggesting the same underlying associative learning principles of observational and direct fear learning, we assume that greater CS + /CS- differences in verbal reports reflect the larger number of learning trials for observers of the calm and fearful demonstrator, as compared to participants of the control condition who began learning that one of the two cues predicts temporary dyspnea only during the direct acquisition phase Interestingly, greater CS + /CS - differences in verbal reports for observers of the calm and fearful demonstrator relative to participants of the control condition resulted from smaller US-expectancy and anxiety in response to CS -, suggesting that the larger number of learning trials seems to have a stronger impact on safety learning in response to CS -. Nevertheless, the transfer of contingency knowledge from observational to direct fear learning for observers of the calm and fearful demonstrator relative to observers of the control condition was likely also facilitated by the contingency interview that was conducted at the end of the observational learning phase. The assessment of contingency ratings is assumed to focus participants’ attention on possible CS-US contingencies, which has been discussed to enhance subsequent fear conditioning [comp. [34], [35]]. Unfortunately, the present design does not allow us to disentangle the individual contribution of each manipulation, i.e., observational learning and the contingency interview. However, we note that exploratory analyses also suggested that CS + /CS - differences in US-expectancy ratings were greater for observers of the fearful relative to the calm demonstrator at the beginning of the direct acquisition phase, which provides another hint that CS + /CS - differentiation was facilitated following observational learning involving fearful as compared to calm demonstrator’s responses.

As expected, and corresponding to subjective ratings, we found that participants of all groups exhibited greater autonomic arousal in response to CS + as compared to CS - throughout direct acquisition, as indicated by greater SCRs and stronger late cardiac deceleration in response to CS + vs. CS -. The emergence of differential fear responses in SCRs across participants of all groups aligns with the results from other interoceptive fear conditioning studies [e.g., 36], reflecting successful learning in a conditioning procedure involving the first-hand experience of an aversive interoceptive stimulus. Correspondingly, we also found greater startle response magnitudes in response to CS + relative to CS - across participants of all groups. Nevertheless, we also found that observers of all groups exhibited a significant potentiation of startle responses towards both CSs as compared to ITI. It is possible that this pattern indicates an overall sensitization of the defensive response system, due to high salience of aversive interoceptive stimuli, such as temporary dyspnea, which may increase the likelihood of fear generalization [37], [38]. At the same time, the CS- may not represent a completely neutral condition, and to some extent, greater startle response magnitudes relative to ITI may also be expected in response to CS- [see also [35], [38]]. In addition, some findings of the complementary analyses are worth noting. First, across participants of all groups, we found a stronger late cardiac deceleration in response to CS + relative to CS -, a response pattern that has been associated with fear-induced bradycardia [15]. Further, we found an increase in respiratory rate and minute ventilation that was more pronounced during the presentation of CS + relative to CS -. While differential changes in respiration were specific for observers of the fearful demonstrator at the end of the observational learning phase, and higher minute ventilation in response to CS + relative to CS - was maintained at the beginning of the direct acquisition phase for these observers, increases in respiratory rate and minute ventilation to CS + relative to CS - emerged throughout the direct acquisition phase for participants of all groups. Previous studies have reported increases in respiratory rate and tidal volume, resulting in greater minute ventilation during the anticipation or imagery of interoceptive threat, which is assumed to reflect defensive respiratory responses [16], [17], [30], [32], [39], [40]. Nevertheless, corresponding to the other subjective and physiological measures, changes in respiratory patterns in response to CS + relative to CS - did not differ between groups at the end of the direct acquisition phase.

Notably, exploratory analyses offered additional insights during the direct acquisition phase. Specifically, we found that observers of the fearful demonstrator exhibited a significantly greater potentiation of startle responses in response to CS + as compared to CS -, while no such difference was found for observers of the calm demonstrator. As the eyeblink startle response constitutes a low-level brain stem reflex that is mediated by the activation of defensive brain networks [32], these findings give a first hint towards greater activation of the defensive circuit for observers of the fearful demonstrator. Previous studies revealed increased defensive mobilization in response to bodily sensations in individuals with high anxiety sensitivity, which is characterized by higher threat expectations in response to bodily sensations [33], [34]. A comparable pattern was also found in individuals with panic disorder as compared to healthy controls, but only for cues predicting the occurrence of interoceptive, but not exteroceptive threats, suggesting that sensitization was specific for threat-relevant cues [35]. Although preliminary in nature, based on the extant literature, greater CS + /CS - differences in observers of the fearful demonstrator may be interpreted as a first hint towards an – at least temporary - sensitization of the defensive response system to cues that have previously been paired with temporary dyspnea for this demonstrator, potentially suggesting that differential learning in startle responses was facilitated for these observers (comp. [27]).

#### 4.1.2 Direct extinction phase

At the beginning of extinction, observers of all groups maintained significantly greater anxiety in response to CS + relative to CS - as indicated by verbal reports. Despite the absence of differences in absolute ratings, observers of the calm and fearful demonstrator reported greater CS + /CS - differences in subjective anxiety relative to participants of the control condition at the beginning of the extinction. As compared to participants of the control condition, that started learning the stimulus contingencies only during direct acquisition, this may be attributed to the larger number of learning trials and additional rehearsal during the contingency interview at the end of the observational learning phase. Moreover, CS + /CS - differences were more pronounced for observers of the fearful demonstrator relative to the control condition at the beginning of the extinction, resulting from smaller US-expectancy in response to CS -, hinting at an additional advantage in differential fear learning that for observers of the fearful demonstrator. Despite descriptive trends we did not find that the course of extinction was modulated by initial observational learning experiences in physiological or subjective measures. While we found that the expression of fear decreased in verbal reports and startle response magnitudes during unreinforced presentations of the CSs, participants of all groups reported greater US-expectancy and anxiety in response to CS + as compared to CS - at the end of direct extinction and maintained greater startle response magnitudes in response to CS + as compared to ITI Moreover, there were no more group differences in cardiac and respiratory measures in response to CS + relative to CS - at the end of the extinction phase, and there were no CS + /CS - differences in SCRs and there was no further decline over the course of the extinction phase. Although the reduction of expressed fear during extinction aligns with our hypothesis and other interoceptive fear conditioning studies [14], [41], hints towards differential responding were still evident in some measures at the end of the extinction, indicating incomplete extinction, which contradicts our hypothesis. However, relative to the large number of observational and direct learning trials, we assume that this is likely to reflect the relatively small number of extinction trials and suggests that future studies should extend the number of trials to fully cover extinction processes.

### 4.2 Theoretical and clinical implications

While we found that verbal reports, SCRs, late cardiac decelerations and changes in respiratory rate showed a largely converging pattern of CS + /CS - differences upon the end of the direct acquisition phase, the interpretation of systematic group differences remains more complex. With our multimodal approach, we aimed to characterize the interaction of different observational learning experiences and direct fear learning across different response systems. We further aimed to evaluate whether observational learning from a fearful demonstrator’s response facilitates subsequent fear learning relative to a calm demonstrator’s response or the absence of prior observational learning experience (control condition). In the present study, startle responses seemed to be the most sensitive physiological index to capture the influence of different prior learning experiences on subsequent direct fear acquisition. Although our findings remain tentative and warrant replication, exploratory analyses suggest that the differential activation of the defensive response system, and consequently, differential fear responses in subcortical structures were facilitated for observers of the fearful demonstrator [35], [42], which may be interpreted as an indicator of sensitization to interoceptive fear conditioning (comp. [10]). The startle eyeblink response constitutes a sensitive measure of fear at the reflex level, indicating defensive mobilization and valence dependent action preparation that is not dependent on cortical processing [37], [43], [44] and contingency knowledge [45]. While the greater potentiation of startle response magnitudes indicate that differential learning might be facilitated on the subcortical level after observational learning from a fearful demonstrator, this pattern was not evident across the other outcome measures. However, in line with Lonsdorf et al. [35], convergence across different response systems is not necessarily expected and different outcome measures are assumed to capture at least partly distinct dimensions of fear, with the potentiation of startle response magnitudes indicating learning on the level of defensive reflex mobilization.

Overall, our findings contribute to the growing body of research highlighting the role of observational learning in the social transmission of fear, as originally proposed by Rachman [8]. Compared to the absence of such learning experiences, we have shown that observing another individual's response to an aversive interoceptive stimulus, i.e., temporary dyspnea, provides important information that can then be transferred to situations in which the observers are directly exposed to the stimuli themselves. In addition, we systematically compared how observational learning experiences interact with subsequent fear learning, providing a first attempt to empirically test the modulating role of different observational learning experiences in the development of pathological fear and anxiety [9]. Our study provides initial evidence that differential learning may be facilitated, after observational learning from a demonstrator responding fearfully to temporary dyspnea. With that, the presents study contributes to the literature by providing a first attempt to complement the existing evidence from retrospective questionnaires studies, revealing a positive association of prior learning experiences involving observation of fearful reactions to bodily symptoms during childhood and the onset of panic disorder during adulthood [13], [46]. Yet, the exploratory and preliminary nature of the present findings limits the extent to which theoretical and clinical implications can be drawn. Moreover, to the best of our knowledge, this was the first study to test the impact of observational learning involving a calm, non-fearful demonstrator’s response to temporary dyspnea. Nevertheless, the findings for the calm demonstrator group remain inconclusive. While the absence of CS + /CS - differences may indicate inhibition of differential learning, for example as a result of lower perceived threat relevance, these results may also imply a stronger tendency towards fear generalization from CS + to CS -, possibly pointing towards higher ambiguity caused by the calm demonstrator’s response (comp. [38]). Unfortunately, the present study does not allow any conclusions about these competing explanations. We hope to encourage future research to examine the underlying mechanisms through which different observational learning experiences interact with fear learning in the future. Overall, future research using larger samples, including subclinical and clinical populations, is needed to draw more robust conclusions regarding the role of observational and direct fear learning in theories on the etiology of pathological fear and anxiety, particularly panic disorder.

### 4.3 Limitations and future directions

Although we believe that our control condition provides a strong comparison of social versus non-social learning experiences, we acknowledge that other control conditions, e.g., a pure control condition without any prior experiences with the visual cues, would have been necessary to determine which mechanisms underlie the observed pattern of results. Moreover, such a control condition would have been useful to minimize interpretational ambiguities, e.g. regarding the possible contribution of latent inhibition [comp. 33] and the potential influence of the contingency interview [comp. 47]. Moreover, we acknowledge that observers of the fearful and the calm demonstrator were depicted with a breathing mask, which itself might be perceived as threatening by the observers. While this setup aligns with other observational fear conditioning studies employing exteroceptive [e.g., 48] or interoceptive stimuli [4], it is possible that this resulted contextually triggered anxiety, which might have reduced the sensitivity to detect group differences based on the demonstrator’s response or resulted in a stronger tendency of fear generalization from CS + to CS - [38]. Although this setup was intentionally chosen to compare different demonstrator’s responses to the same aversive respiratory stimulation, future studies could employ a control group in which the demonstrator is depicted with a face mask, but no inspiratory resistive loads are applied to the demonstrator (unreinforced presentations, [comp. 6]). We assume that this would be a useful approach to isolate possible effects of an aversive context (as signaled by the face mask) from the demonstrator’s actual response to acute respiratory threat.

#### 4.3.1 Robustness of effects and constraints on generality

Although differential fear learning during the direct acquisition phase, reflected in stronger fear responses to CS + relative to CS -, was evident across several subjective (US-expectancy and anxiety ratings) and physiological (SCRs, startle response magnitudes, late cardiac deceleration, and respiratory rate) measures, evidence for group differences was more limited. Group differences emerged at the end of the observational learning phase and at the beginning of the direct acquisition phase, but effects in the predicted direction were restricted to verbal reports. For our primary physiological outcome measure, we identified a medium-sized Stimulus × Group interaction for startle response magnitudes during direct acquisition phase (*η*_*p*_^*2*^=.08, see also Table 1), which however, did not reach statistical significance. While post-hoc power analyses suggested that the achieved power was sufficient to detect an effect of this magnitude, statistical power was limited for detecting more subtle differences, such as changes in startle response magnitudes throughout learning, i.e., the direct acquisition phase (Block x Stimulus x Group: *η*_*p*_^*2*^ =.02, 1 − β =.65, see also Table 1). Finally, some of our findings suggest that the effects seem to depend on specific boundary conditions, such as the comparison of the observational learning groups only, underscoring that the findings of the present study should be considered preliminary. Although we adhered to our pre-registered target sample size, we emphasize that replications with larger, more diverse samples are needed to draw stronger conclusions about the robustness of the effects across different outcome measures.

Further, we note that the video material depicted only one male confederate. As observational learning has been found to be sensitive to characteristics of the demonstrator, such as the perceived similarity [2] this substantially constrain the generality of the findings. Moreover, although participation in this study was not constrained beyond the pre-defined exclusion criteria, post-hoc inspection of the sample demographics indicated that a more diverse sample would have been desirable. We therefore encourage future studies to employ multiple demonstrators and to also recruit more diverse samples to examine variation in demonstrator characteristics and their interaction with participant characteristics, which would help delineate how and under what boundary conditions observational learning may contribute to the etiology of pathological fear and anxiety.

## 5 Conclusions

Taken together, the present study provides an experimental analog to systematically examine the role of observational learning experiences in the development of pathological fear and anxiety, revealing observational learning of a fearful demonstrator as potential pathway that may facilitate differential fear learning of interoceptive threat, which warrants further investigation. While verbal reports and startle responses lend preliminary support for the potential role of observational learning in shaping future acquisition of fear of bodily symptoms, in order to enhance clinical translations, future research is needed to understand how observational learning interacts with direct fear learning in shaping the risk for the development of pathological fear and anxiety.

## CRediT authorship contribution statement

**Carlotta Paulina Albert**: Writing – review & editing, Writing – original draft, Methodology, Investigation, Formal analysis, Conceptualization. **Christoph Benke**: Writing – review & editing, Methodology, Conceptualization, Project administration. **Christiane A. Melzig**: Writing – review & editing, Methodology, Conceptualization, Supervision, Project administration, Funding acquisition.

## Funding

This work was supported by the DYNAMIC center, which is funded by the LOEWE program of the Hessian Ministry of Science and Arts (Grant Number: LOEWE1/16/519/03/09.001(0009)/98) and by the Deutsche Forschungsgemeinschaft (DFG, German Research Foundation), Project-ID 422744262, TRR 289 (Gefördert durch die Deutsche Forschungsgemeinschaft (DFG) – Projektnummer 422744262 – TRR 289).

## Declaration of Competing Interest

The authors declare that they have no known competing financial interests or personal relationships that could have appeared to influence the work reported in this paper.

## Footnotes

Appendix A Supplementary data associated with this article can be found in the online version at doi:10.1016/j.xjmad.2026.100199.
