# Supplementary material for: How observing others influences the subsequent acquisition of fear of bodily sensations
Source: J Mood Anxiety Disord. 2026 Aug 4;15:100199. doi: 10.1016/j.xjmad.2026.100199 (PMC13495619; doi:10.1016/j.xjmad.2026.100199)
Supplement: Supplementary file 1 [file mmc1.docx]

# A. Supplementary Methods

## 1. Sample

Following a telephone screening, participants with cardiovascular, respiratory (e.g., asthma, cold), neurological (e.g., epileptic or apoplectic seizures, multiple sclerosis) conditions, regular medication use, or ongoing psychotherapeutic treatment were excluded. Additional exclusion criteria were impaired hearing or color vision and reported pregnancy. We also excluded participants reporting prior participation in studies conducted in our lab employing interoceptive fear conditioning. For all participants, sex assigned at birth corresponded to the self-reported gender identity. *N*= 88 individuals participated in the study. Prior to analyses, we excluded participants because they did not experience temporary dyspnea during the direct acquisition phase (*n*= 6), violation of the experimental protocol (*n*= 2) or technical failures (*n* = 2), termination before completion of the experiment (*n* = 3), and one person who reported sleeping during the observational learning phase (*n*= 1).

| **Table S1.** Means and Standard Deviations of Self-reported Questionnaire Measures. | | | | |
| --- | --- | --- | --- | --- |
|  | Groups | | | Test |
|  | Control Condition  (*n*= 25) | Calm Demonstrator  (*n*= 25) | Fearful Demonstrator  (*n*= 24) |  |
| CLQ-SF  [0-56] | 10.04 (7.03) | 8.04 (6.83) | 9.96 (8.60) | *F*(2,71)= 0.56, *p*= .571 |
| ASI-3  [0-72] | 25.90 (10.80) | 23.80 (10.80) | 26.40 (9.10) | *F*(2,71)= 0.44, *p*= .647 |
| LHQ-Obs  [0-9] | 0.95 (1.25) | 1.22 (1.50) | 1.16 (1.62) | *F*(2,71)= 0.24, *p*= .785 |
| Age | 24.3 (5.12) | 24.6 (4.52) | 24.3 (4.86) | *F*(2,71)= 0.02, *p*= .977 |
| Gender  [f/m] | 9/15 | 11/14 | 11/14 | *χ²*(2)= 0.28, *p* = .869 |
| *Note.* Means and Standard Deviations (SD) for CLQ-SF: Claustrophobia Questionnaires Suffocation Fear Subscale, ASI-3: Anxiety Sensitivity Index, LHQ – Obs: Learning History Questionnaire (Observation of Parents Sick Role Behavior or Panic Symptoms x Frequency of observation), and age. Possible questionnaire score ranges are reported in parentheses. | | | | |

## 2. Material

### Video Material

During the video shooting, the demonstrator was given additional visual information to secure perfect timing of the displayed respiratory and emotional response as planned for video timing. This visual info was later edited out of the videos thus only the geometrical shape assigned to CS+ was visible on the screen. The demonstrator was also instructed to amplify his facial expression so that the respiratory and emotional responses could be decoded easily by any video observer and to reduce ambiguity (cf. [1]). For each trial type, the same video sequences were used and repeatedly presented throughout the observational learning phase according to the defined number of trials, as described below. The confederate demonstrator was wearing the same headphones, silicone mask and sensors that were used for the participants, mirroring the experimental setup of the study participant. The stimulus material used for observational learning can be made available upon request.

### Breathing Circuit and Unconditioned Stimulus

All participants were fitted with an appropriately sized soft silicone face mask (7400 series; Hans Rudolph, Inc., Kansas City, MO), tightly covering mouth and nose. The mask was connected to a tube with a mounted flow sensor at the mouth port of a two-way y-shaped non-rebreathing valve (no. 2630; Hans Rudolph, Inc.). The expiratory port enabled unrestricted expiration throughout the experiment. A plastic tube (length: 2.75 m; diameter: 35 mm) was mounted to the inspiratory port of the valve, bridging the distance to the adjacent control room, where the tube was connected to the common port of a Five-Way Gatlin-Shape™ Inflatable-Balloon-Type™ valve (2440 series, Hans Rudolph, Inc.). A pneumatic controller (2430 series, Hans Rudolph, Inc.) with 4 experimental ports was used, of which one was connected to nylon flow resistor of linear type (7100 series, Hans Rudolph, Inc.), allowing for the application of inspiratory resistive loads (IRLs). All other ports remained unrestricted. Switches between restricted and unrestricted breathing were controlled by Presentation Software (Version 23.0, Neurobehavioral Systems, Inc., Berkeley, CA, [www.neurobs.com](http://www.neurobs.com)).

IRL intensity was selected to elicit moderate dyspnea and was determined based on prior studies using the same respiratory setup [2]. We opted for a standard load intensity to ensure that all participants started naively, i.e., without any prior experience with temporary dyspnea, into observational and direct acquisition. This was intended to prevent any potential interference from prior experience on the effects of observational learning and subsequent direct fear acquisition.

### ***Subjective*** Ratings: Perception of the Demonstrator

*Perceived Anxiety or Discomfort in the Demonstrator*

Participants reported the perceived anxiety or discomfort of the demonstrator during the presentation of observational CS+ and observational CS- using a 6-point Likert scale (1 *“no anxiety or discomfort at all”* – 6 *“extreme fear or discomfort”*).

#### Observed Panic Symptoms

Participants reported which symptoms they had observed in the demonstrator during the presentation of the two CSs by selecting from a list of nine panic symptoms [3].

### Subjective Ratings: Participants’ Experience

#### Experienced Valence Ratings

Participants used a 9-point Likert scale to provide ratings about the experienced arousal experienced valence (1 *“pleasant”* – 9 *“unpleasant”*) in relation to observational CS+ and observational CS-.

### Questionnaires

#### Contingency Awareness Interview

We assessed participants’ awareness of the contingency between the observational CSs and the observational US (temporary dyspnea) for the demonstrator via interview. Questions addressed the frequency of US occurrences and whether participants knew when temporary dyspnea was induced for the demonstrator, i.e., awareness of the association of one geometrical shape and the US (*“yes” – “no”*)*.* Moreover, for each geometrical shape, participants were asked whether the presentation was followed by temporary dyspnea for the demonstrator (“*rather yes*” – “*rather no*”), and with which likelihood it was predicted by the geometrical shape (*0% - 100%*).

#### Claustrophobia Questionnaire

The Suffocation Fear subscale of the Claustrophobia Questionnaire (CLQ; [4]) includes 14 items rated on a 5-point Likert scale ranging from 0 to 4 (0 *“Not at all anxious” -* 4 = *“Extremely anxious”),* assessing participants’ experienced anxiety in situations related to suffocation fear (e.g., “swimming while wearing a nose plug”, „trying to catch your breath during vigorous exercise “).

#### Anxiety Sensitivity Index

Anxiety Sensitivity Index (ASI; [5]) is used to assess participants’ fear of anxiety-related symptoms. Participants respond to 18 items using a 5-point Likert scale ranging from 0 – 4 (0 *“very little”* – 4 *“very much”*).

#### Learning History Questionnaire

The Learning History Questionnaire (LHQ; [6]) retrospectively assesses learning experiences related to bodily symptoms. We used the “Observation of Parental Sick-Role Behavior/Panic Symptoms” subscale, which includes 20 items rated on a 4-point Likert scale (0 *“never”*; 1 “*seldom”*; 2 *“occasionally”*; 3 *“often”*). An additional item assesses the frequency of parental panic-related symptoms (e.g., dizziness, shortness of breath), again using a 4-point Likert scale (0 *“never”*; 1 *“once or twice a year”;* 2 *“three to six times a year”*; 3 *“more than six times a year”*). An index of observational learning experiences is computed by multiplying the mean parental sick-role behavior score with the frequency of panic-related symptoms (see [6]).

## 3. Physiological Recordings

### Electromyography

The eyeblink component of the startle response was measured by continuously recording electromyographic (EMG) activity over the *orbicularis oculi* muscle below the left eye using two electrolyte-filled Ag/AgCl miniature surface electrodes. The signal was filtered using a 10 Hz high-pass and 500 Hz low-pass filter. Digital sampling was set to 2000 Hz. The EMG signal was amplified by factor 5000 using the EMG100C electromyographic amplifier (Biopac Systems, Inc., Goleta, California, USA).

### Electrodermal Activity

Electrodermal activity was recorded from the hypothenar eminence on the palm of the participants’ non-dominant hand using two Ag/AgCl standard electrodes (8 mm diameter) filled with a 0.05 M sodium chloride electrolyte medium. A constant DC voltage of 0.5 V was applied across electrodes by an EDA100C electrodermal activity amplifier (Biopac Systems, Inc., Goleta, California, USA). Signals were filtered with a 10 Hz low-pass filter and recorded with a resolution of 5µS/V. The amplified signal was sampled continuously at a rate of 2000 Hz.

### Electrocardiogram

ECG was measured with electrolyte-filled Ag/AgCl standard electrodes (8 mm diameter) attached in an Einthoven-II configuration. The raw ***s***ignal was amplified and filtered with a 35 Hz low-pass filter and a 1 Hz high-pass filter using an ECG100C amplifier (Biopac Systems, Inc., Goleta, California, USA) and the sampling rate was set to 2000 Hz.

### Respiratory Measures

Respiratory flow, mouth pressure, and fractional CO2 and O2 were continuously recorded during the experiment using a modified ZAN 600 system (nSpire Health, Inc., Oberthulba, Germany).

## 4. Response Definition and Data Reduction

### Electromyographic Activity

The raw EMG signal was filtered off-line with a 60 Hz high-pass filter, a 400 Hz low-pass filter, rectified and smoothed using a first-order low-pass filter with a time constant of 10 ms. Response definition was performed using a computer program [7], identifying the latency of blink onset and peak (ms) and the resulting amplitude. The signal was visually inspected for artefacts and onset and peak corrected, when necessary. In case no blinks were detected, magnitude was set to zero (“nonresponse”). Trials with excessive recording artifacts were set as missing.

### Skin Conductance Response

SCRs were manually scored using a computer program [7]. When no response was detected, amplitudes were set to zero (“nonresponse”). Trials with excessive recording artifacts were set as missing.

### Electrocardiogram

ANSLAB 2.4. (Autonomic Nervous System Laboratory, University of Basel, Switzerland) was used for automatic R-wave detection. The ECG signal was visually inspected, and R wave triggers were manually corrected, if necessary. Recording windows with excessive movement artifacts were excluded and set to missing. Interbeat-intervals were calculated and converted to beats per minute as a measure of heart rate. For each half-second a change score was calculated by subtracting the mean from a 1 s baseline before CS-onset for each trial. Change scores were averaged for each CS-type and across four trials for each block of the observational learning and the direct acquisition phase and across blocks of three trials for each block during direct extinction phase. In addition, we calculated heart rate components D1 (early cardiac deceleration), as the minimum heart rate during the first 2 s after CS-onset, A (cardiac acceleration), as the maximum heart rate after D1 and within the first 5 s after CS-onset, as well as D2 (late cardiac deceleration), as the minimum heart rate in the interval between A and US onset [8]. A and D2 component were corrected for the influence of the preceding components by subtraction (A= A-D1 and D2= D2-A) [9].

### Respiratory Measures

Data was visually inspected and were set to missing in the case of artifacts, such as coughing. Maximal mouth pressure during inspiration was automatically identified and corrected manually when necessary, using ANSLAB 2.4. Finally, mouth pressure, respiratory rate, tidal volume, and minute ventilation were exported in weighted 1s means.

We calculated a change score by subtracting the mean for each second from a 3 s baseline before CS-onset. Differences scores were averaged for each CS-type across blocks of four trials in the observational learning and the direct acquisition phase and across blocks of three trials during the direct extinction phase.

### Panic Symptoms

We counted the number of participants who reported to have observed at least one respiratory symptom (i.e., shortness of breath or difficulty breathing, feeling of choking, or chest pain or discomfort) in the demonstrator for observational CS+ and observational CS-. Then, we calculated the proportion of participants who reported at least one respiratory symptom within the calm and fearful demonstrator group.

### US-Expectancy and Anxiety Ratings

We averaged subjective anxiety and US-expectancy ratings across blocks of four trials for the acquisition phase, and across blocks of three trials for the extinction phase.

## 5. Procedure

Upon arrival in the laboratory, participants read and signed the informed consent forms, including data protection regulations. Then, they entered the experimental room, where electrodes were attached, an appropriately sized silicon face mask was fitted to participants’ head and connected to the breathing circuit. Participants were put on headphones. Participants were randomly assigned to one out of the three experimental groups. The geometrical shapes assigned to CS+ and CS- were counterbalanced between participants. The CSs were presented in a pseudo-randomized order with the restriction of no more than two trials of the same type (i.e., CS+ vs. CS-) being presented in consecutive order. The order of stimulus presentation varied between the experimental phases. First, participants were instructed for the *habituation* and the *observational learning phase*. Participants were told that they would watch a video and that their only task was to follow it carefully. They were not given any information about the content of the video. Although participants were already wearing the same experimental equipment as the demonstrator, they were *not* informed that they would complete the same procedure later in the experiment. Finally, they were told that loud noises can occur, that can be ignored. During observational learning, we opted for a 75% reinforcement rate, to use the same reinforcement rate as during the subsequent direct acquisition phase. The reinforcement schedule was selected to prolong potential habituation effects towards the US during the direct acquisition phase (cf. Lonsdorf et al., 2017). During the startle response *habituation* *phase* a black screen was presented for 100 s, and six startle probes were delivered. Then, participants completed the *observational learning*, followed by a contingency interview and ratings of the perceived intensity and unpleasantness of temporary dyspnea for the demonstrator, as well as the observed panic symptoms using an online form. Afterwards, participants were told that they would now go through the same experimental procedure as previously shown in the video, and that they would see the same geometrical shapes. They were informed that temporary dyspnea could be induced and they were reminded that loud noises could be ignored. They were *not* given any further information about the stimulus contingency or the duration and intensity of the induced temporary dyspnea. After completion of the *direct acquisition*, participants were instructed that the same geometrical shapes would be presented to them, that temporary dyspnea could be induced, and that loud noises could be ignored. Then, participants completed the *direct extinction*. Finally, sensors were removed, and participants answered several questionnaires using the online rating form. Before leaving, participants were debriefed on the study’s goals and received financial compensation or course credits.

# B. Supplementary Analyses

### Perception of Respiratory Symptoms

For observers of both, the calm and fearful demonstrator group, the proportion of participants that perceived at least one respiratory symptom in the demonstrator was significantly greater during observational CS+ as compared to observational CS- trials, *χ²*s > 6.75, *p*s < .009. The groups did not differ in the proportion of participants who perceived at least one respiratory symptom during CS+ trials, *χ²*(1) = 1.57, *p* = .210, or CS- trials, *χ²(*1) = 0.04, *p* = .851. See Table S2 for the exact proportions.

## Valence Ratings after Observational Learning

Observers of the calm and fearful demonstrator reported greater CS+/CS- differences in valence ratings as compared to participants of the control condition, *F*(2,71)*_StimulusxGroup_*= 7.93, *p*< .001, *η_p_^2^*= .18, 95%CI [0.06, 1.00]. Significantly greater CS+/CS- differences for observers of the fearful demonstrator as compared to participants of the control condition, *t*(71)*_Fearful>Control_*= 3.98, *p*< .001, resulted not only from lower reported displeasure in response to CS-, *t*(142)*_Fearful>Control_*= -2.54, *p*= .033, but also greater reported displeasure in response to CS+, *t*(142)*_Fearful>Control_*= 2.96, *p*= .012.

| **Table S2.** Differences in CS-Ratings (CS+ - CS-) after the Observational Learning Phase. | | | | | | | | | |
| --- | --- | --- | --- | --- | --- | --- | --- | --- | --- |
|  | Experimental Condition | | | | | | | | |
|  | Calm Demonstrator | | | Fearful Demonstrator | | | Control Condition | | |
| Measure | *M* (*SD*) | |  | *M* (*SD*) | |  | *M* (*SD*) | |  |
|  | CS+ | CS- | *t(24)* | CS+ | CS- | *t(23)* | CS+ | CS- | *t(24)* |
| Valence  [1-9] | 5.24 (1.61) | 3.96 (1.93) | 2.59* | 6.12 (1.45) | 3.62 (1.56) | 5.55*** | 4.80 (1.35) | 4.76 (1.42) | 0.11 |
| Arousal  [1-9] | 4.8 (1.94) | 3.44 (1.66) | 2.90** | 5.75 (2.09) | 2.96 (1.76) | 4.42*** | 4.20 (1.77) | 4.2 (1.85) | 0.16 |
| Resp. Symptoms | 20/25 | 10/25 |  | 23/24 | 8/25 |  | - | - |  |
| *Note.* At the end of the observational learning phase, participants were asked to rate their experience in relation to the observational CSs presented in the videos. Range of possible scores are reported in parentheses, with larger scores indicating more displeasure and higher arousal. Resp. Symptoms: proportion of participants that perceived at least one respiratory symptom in the demonstrator. * *p* < .05; ** *p* < .01; *** *p* < .001 | | | | | | | | | |

## Anxiety Ratings

### Observational Learning

Observers of the calm and fearful demonstrator reported greater anxiety or discomfort in response to CS+ as compared to CS- at the end of observational learning, all *t*s_CS+>CS-_> 3.52, all *p*s< .001, while no CS+/CS- difference was evident in participants of the control condition, *t*(71)*_CS+>CS-_*= -0.84, *p*= .405, *F*(2, 71)*_StimulusxGroup_*= 12.64, *p*< .001, *η_p_^2^*= .26, 95% CI [0.12, 1.00] (see Figure A1, Panel A). While magnitude of CS+/CS- differences did not differ between observers of the calm and fearful demonstrator, *t*(71)*_Fearful>Calm_*= 1.93, *p*= .172, CS+/CS- differences were greater for both groups as compared to participants of the control condition, all *t*s_Fearful,Calm>Control_> |-3.08|, all *p*s< .009. This resulted from participants of the control condition reporting smaller anxiety or discomfort in response to CS+, all *t*s*_Cal,Fearful>Control_*> 2.77, all *p*s< .018, while there were no group differences in response to CS-, all *t*s*_Calm,Fearful>Control_* < 2.18, all *p*s < .093.

### Acquisition

Across participants of all groups, CS+/CS- differences in subjective anxiety increased during acquisition, *F*(2, 284.0)*_BlockxStimulus_*= 4.76, *p*= .009, *η_p_^2^*= .03, 95% CI [0.00, 1.00], resulting from a decrease in fear responses to CS-, *t*(229)*_A1>A3_*= 6.08, *p*< .001, while there was no significant change in reported anxiety in response to CS+, *t*(229)*_A1>A3_*= 1.86, *p*= .154. (see Figure A1, Panel B). Although participants of all groups reported greater subjective anxiety in response to CS+ as compared to CS- during acquisition, all *t*s*_CS+>CS-_*> 5.37, all *p*s< .001, the magnitude of CS+/CS- differences differed between groups, *F*(2,284.0)*_StimulusxGroup_*= 16.21, *p* <.001, *η_p_^2^*= .10, 95% CI [0.05, 1.00] (see Figure 3, Panel B). While observers of both the calm and fearful demonstrator reported greater CS+/CS- differences in subjective anxiety as compared to participants of the control condition, all *t*s*_Fearful,Calm>Control_*>4.48, all *p*s< .001, follow-up comparisons of absolute anxiety ratings revealed that observers of the fearful demonstrator group reported significantly smaller anxiety in response to CS- as compared to participants of the control condition, *t*(88.7)*_Fearful>Control_*= -2.58, *p*= .031, while all other comparisons between groups did not reach significance, all *t*s< 1.85, all *p*s> .158. The magnitude of CS+/CS- differences did not differ between observers of the calm and fearful demonstrator group, *t(*284)*_Fearful>Calm_*= -0.84, *p*= .789. The three-way interaction did not reach significance, *F*(4, 284.0)*_BlockxStimulusxGroup_*= 0.83, *p*= .510, *η_p_^2^*= .01, 95% CI [0.00, 1.00].

### Extinction

Observers of both, the calm and fearful demonstrator group reported significantly greater CS+/CS- differences in subjective anxiety ratings as compared to participants of the control condition at the beginning of extinction, all *t*s*_Fearful,Calm>Control_*> 2.54, all *p*s< .038, while CS+/CS- differences no longer differed between groups at the end of the extinction phase, all *t*s*_Fearful,Calm>Control_*< 1.46, all *p*s> .396, *F*(2, 71)*_TimexStimulusxGroup_*= 6.16, *p*= .003, *η_p_^2^*= .51, 95% CI [0.00, 1.00] (see Figure A1, Panel B). CS+/CS- differences did not differ between observers of the calm and fearful demonstrator group during extinction, all *t*s*_Fearful>Calm_*< 1.13, all *p*s > .596. Despite greater CS+/CS- differences during the first block of trials for observers of the calm and fearful demonstrator as compared to participants of the control condition, the comparison of absolute anxiety ratings revealed no significant differences in anxiety ratings between groups in response to either CS+ or CS-, all *t*s< 2.26, all *p*s> .068.

**Figure A1.** Subjective anxiety ratings at the end of the observational learning phase and during direct acquisition and extinction. CS= conditioned stimulus, A1-A3= blocks of trials during acquisition, averaged across blocks of four trials. E1-E2= blocks of trials during extinction, averaged across three trials. Mean responses are depicted for each experimental group, with error bars indicating the standard error of the mean.

### Heart Rate Observational Learning

The initial cardiac deceleration, i.e., the D1-component, did not change during observational learning, all *F*s< 2.39, all *p*s> .100. We found a significant decrease in cardiac acceleration, i.e., the A-component, from the first to the last block of the observational learning phase, *F*(2, 320.0)*_Block_*= 6.83, *p*=.001, *η_p_^2^* = .04, 95%CI [0.01, 1.00], that did not differ between stimuli or groups, all *F*s< 1.15, all *p*s> .329. We also detected a significant decrease in late cardiac deceleration, i.e., the D2-component, throughout the observational learning phase, *F*(2, 320.0)*_Block_*= 6.75, *p*=.001, *η_p_^2^* = .04, 95%CI [0.01, 1.00], which did not differ between stimuli or groups, all *F*s< 1.40, all *p*s> .248 (see Figure A2, Panel A). Change scores for each half-second during CS-presentation are presented in Figure A3, Panel A.

### Acquisition

For participants of all groups, initial cardiac decelerations decreased from the first to the last block of the direct acquisition phase for participants of all groups, *F*(2,315.0)*_Block_* = 5.73, *p*= .004, *η_p_^2^* = .04, 95%CI [0.01, 1.00]. No systematic CS+/CS- differences in initial cardiac decelerations emerged throughout the direct acquisition phase, *F*(2,315.0)*_BlockxStimulus_*= 1.14, *p*= 0.321. Although we detected a significant three-way interaction, *F*(4, 315.0)*_BlockxStimulusxGroup_*= 2.51, *p*= .042, *η_p_^2^* = .03, 95%CI [0.01, 1.00], this resulted from small differences in initial cardiac deceleration for observers of the calm demonstrator, exhibiting a greater initial cardiac deceleration in response to CS+ relative to CS- during the second block of trials, *t*(315)*_CS+>CS_*_-_= -2.04, *p*= .042. Cardiac acceleration did not change throughout the acquisition phase and did not differ between stimuli or groups, all *F*s< 1.16, all *p*s> .329. Late cardiac deceleration was overall more pronounced in response to CS+ as compared to CS- during acquisition, *F*(1, 316.0)*_Stimulus_*= 11.11, *p*< .001, *η_p_^2^* = .03, 95%CI [0.01, 1.00], which did not change throughout learning and did not differ between groups, all *F*s< 1.252, all *p*s> .289 (see Figure A2, Panel B). Change scores for each half-second during CS-presentation are presented in Figure A3, Panel B.

### Extinction

Initial cardiac decelerations did not change throughout the direct extinction phase, *F*(1,80.6)*_Block_*= 3.02, *p*= .086, and did not differ between stimuli and groups, all main effects and two-way interactions, all *F*s< 0.87, all *p*s> 0.42. We found a significant three-way interaction emerged, *F*(2,120.0)*_BlockxStimulusxGroup_* = 3.27, p= .042, *η_p_^2^* = .05, 95%CI [0.01, 1.00], that was driven by observers of the fearful demonstrator, exhibiting a slightly stronger decline in initial cardiac deceleration in response to CS- in the first as compared to the second block of the extinction phase *t*(126)*_E1>E2_*= -2.55, *p*= .012. Cardiac acceleration did not change throughout extinction and was not modulated by stimulus or experimental group, all *F*s< 2.53, all *p*s> .088. Throughout the direct extinction phase, late cardiac decelerations did not differ in response to CS+ as compared to CS- for participants of all groups, *F*(2, 122.4)*_BlockxStimulusxGroup_*= 0.222, *p*= .801, *η_p_^2^*= .01, 95%CI [0.00, 1.00] (see Figure A2, Panel C). Yet, we found a significant two-way interaction, *F*(2, 62.5)*_BlockxGroup_* = 4.49, *p*= .015, *η_p_^2^* = .05, 95%CI [0.01, 1.00], resulting from observers of the fearful demonstrator and participants of the control condition, who exhibited an overall decrease in late cardiac deceleration from the first to the last block of the extinction phase, all *t*s*_E1>E2_* > 2.26, all *p*s< .027. Change scores for each half-second during CS-presentation are presented in Figure A3, Panel C.

 ***Figure A2.*** For each experimental group and phase, mean changes in HR during presentation of the CSs (conditioned stimuli) are depicted in bpm for each heart rate component: D1 = initial cardiac deceleration. A1 = mid-interval cardiac acceleration. D2 = late cardiac deceleration. Observational learning (O1-O3: average across blocks of four trials), acquisition (A1-A3: average across blocks of four trials), and extinction (E1-E2: average across blocks of three trials). Control= Control Condition, Calm= Calm Demonstrator, Fearful= Fearful Demonstrator. Error bars represent the standard error of the mean.

***Figure A3.*** For each experimental group and phase, mean changes in HR during presentation of the CSs (conditioned stimuli) are depicted in bpm during the 8s of CS-presentation. Heart rate components are annotated: D1 = initial cardiac deceleration. A1 = mid-interval cardiac acceleration. D2 = late cardiac deceleration. Observational learning (O1-O3: average across blocks of four trials), acquisition (A1-A3: average across blocks of four trials), and extinction (E1-E2: average across blocks of three trials). Error bars represent the standard error of the mean.

## Respiratory Measures

### Observational Learning Phase

#### Respiratory Rate

During the observational learning phase, changes in respiratory rate did not differ in response to CS+ relative to CS- for participants of the control condition, *F*(1,2745)*_BlockxStimulus_*= 0.08, *p*= .928, while significant CS+/CS- differences emerged for observers of the calm and fearful demonstrator, all *F*s*_BlockxStimulus_*> 5.60, all *p*s< .004, *F*(4, 2745)*_BlockxStimulusxGroup_*= 3.89, *p*= .004, *η_p_^2^*< .01, 95% CI [0.00, 1.00]. Specifically, at the end of the observational learning phase, observers of the fearful demonstrator exhibited a higher respiratory rate in response to CS+ relative to the CS-, *t*(2745)*_CS+>CS-_*= 4.43, *p*< .001, while observers of calm demonstrator showed an opposite pattern, i.e., lower respiratory rate in response to CS+ as compared to CS-, *t*(2745)*_CS+>CS-_*= -4.74, *p*< .001 (see Figure A4, O1-O3). The four-way interaction did not reach significance, *F*(28, 2745)*_TimexBlockxStimulusxGroup_*= 0.54, *p*= .978.

#### Tidal Volume

Tidal volume decreased relative to baseline during CS-presentation 4 – 8 s after CS-onset, all *t*s> 3.04, all *p*s< .049, *F*(7,2684)*_Time_*= 6.03, *p*< .001, *η_p_^2^*= .02, 95% CI [0.01, 1.00], and this effect did not differ between stimuli and groups, all *by-stimulus* and *by-group* interactions, all *F*s< 1.20, all *p*s> .271. Moreover, we found a significant three-way interaction, *F*(4, 2684)*_BlockxStimulusxGroup_*= 2.80, *p*= .025, *η_p_^2^*< .01, 95% CI [0.00, 1.00], that was driven by observers of the control condition, who exhibited higher tidal volume in response to CS+ relative to CS- during the second block of trials, *t*(93.4)*_CS+>CS-_*= 2.58, *p*= .012, while changes in tidal volume did not further differentiate between CS+ and CS- tidal volume for participants of any group throughout the observational learning phase, all *t*s*_CS+>CS-_* < 1.31, all *p*s> .191. The four-way interaction did not reach significance, *F*(28, 2684)*_TimexBlockxStimulusxGroup_*= 0.36, *p*= .999 (see also Figure A5, O1-O3).

#### Minute Ventilation

Minute ventilation decreased relative to baseline during CS-presentation 3 – 8s s after CS-onset, all *t*s> 3.45, all *p*s< .013, *F*(7,2684)*_Time_*= 16.87, *p*< .001, *η_p_^2^*= .04, 95% CI [0.03, 1.00], which did not differ between stimuli and groups, all *F*s< 0.82, all *p*s> .570 (see Figure A6, O1-O3). We detected a significant three-way interaction, *F*(4,2792.0) *_BlockxStimulusxGroup_*= 2.53, *p*= .039, *η_p_^2^*< .01, 95% CI [0.00, 1.00], resulting from participants of the control condition, who exhibited higher minute ventilation during CS+ relative to CS- during the second block of trials, *t*(87.7)*_CS+>CS-_*= 2.95, *p*= .004. Changes in minute ventilation did not differentiate in response to CS+ relative to CS- for observers of the calm and fearful demonstrator during the observational learning phase, all *F*s*_Stimulus_*< 1.68, all *p*s> .200, and all *F*s*_BlockxStimulus_*< 2.18, all *p*s> .060. The four-way interaction did not reach significance, *F*(28,2684)*_TimexBlockxStimulusxGroup_*= 0.62, *p*= .943.

***Figure A4.*** Mean changes in respiratory rate (breaths per minute) for each experimental group and phase. US-onset for the demonstrator (observational learning) and participants (direct acquisition) 8s after CS-onset. O1-O3: Observational learning, A1-A3: Direct acquisition phase, E1-E2: Direct extinction phase. O1-O3/ A1-A3: average across blocks of four trials. E1-E2: average across blocks of three trials. Error bars represent the standard error of the mean.

### Direct Acquisition Phase

To ensure successful application of IRLs during acquisition in adherence with the interoceptive conditioning protocol, we analyzed changes in mouth pressure averaged for the 8s of CS-presentation and during US presentation (15s). We found that mouth pressure was higher during US-presentation (*M*= 10.03, *SD*= 10.05) as compared to the presentation of CS+ (*M*= 2.05, *SD*= 3.26) and CS- (*M*= 1.47, *SD*= 2.09), respectively. The successful manipulation was substantiated by a significant main effect of stimulus type, *F*(2,112)*_Stimulus_* = 152.29, *p* <.001, *η_p_^2^* = .73, 95%CI [0.66, 1.00], and this effect did not differ between experimental groups, *F*(4,112)*_StimulusxGroup_*= 0.40, *p* = .811.

#### Respiratory Rate

During the direct acquisition phase, we found a stronger increase in respiratory rate in response to CS+ as compared to CS- 6-8s after CS-onset, all *t*s*_CS+>CS-_* > 2.63, all *p*s< .009, *F*(7, 2684)*_TimexStimulus_*= 4.65, *p*< .001, *η_p_^2^*= .01, 95%CI [0.00, 1.00], and CS+/CS- differences increased over the course of the direct acquisition phase, *F*(2, 2684)*_BlockxStimulus_*= 4.65, *p*< .001, *η_p_^2^*< .01, 95%CI [0.00, 1.00].These patterns did not differ between groups, all *by-group* interactions, all *F*s< 1.33, all *p*s> .256 (see Figure A4, A1-A3).

#### Tidal Volume

While tidal volume was slightly reduced relative to baseline 3 s after CS-onset, *t*(2684)= 3.08, *p*= .044, tidal volume increased 6 – 8 s after CS-onset, all *t*s> 3.37, all ps< .005, *F*(7, 2684)*_Time_*= 30.62, *p*< .001, *η_p_^2^*= .07, 95%CI [0.06, 1.00]. Again, we detected a significant three-way interaction, *F*(4,2684)*_BlockxStimulusxGroup_*= 3.11, *p*= .014, *η_p_^2^*< .01, 95%CI [0.00, 1.00], resulting from observers of the calm demonstrator, exhibiting reduced tidal volume in response to CS- relative to CS+ during the first block of trials, *t*(92.3)_CS+>CS-_= 2.13, *p*= .036, while changes in tidal volume did not further differentiate between CS+ relative to CS- for participants of all groups throughout the direct acquisition phase, all *t*s*_CS+>CS-_*< 1.68, all *p*s> .097. The four-way interaction did not reach significance, *F*(28,2684)*_Timex_*_BlockxStimulusxGroup_= 0.98, *p*= .498 (see Figure A5, A1-A3).

#### Minute Ventilation

During CS-presentation, we found an increase in minute ventilation 5 – 8 s after CS-onset, all *t*s> 4.85, all *p*s< .001, that was more pronounced in response to CS+ relative to CS-, *F*(7,2684)*_TimexStimulus_*= 4.63, *p*< .001, *η_p_^2^*= .01, 95%CI [0.00, 1.00]. Although we found a significant three-way interaction, *F*(4,2684)*_BlockxStimulusxGroup_*= 3.27, *p*= .011, *η_p_^2^<* .01, 95%CI [0.00, 1.00], this interaction was not driven by systematic changes in response to CS+ relative to CS- for participants of any group, all *t*s*_CS+>CS-_*< 1.92, all *p*s> .058, but resulted from observers of the fearful demonstrator, who exhibited higher minute ventilation during CS+ relative to observers of the calm demonstrator during the first block of the acquisition phase, *t*(65.2)*_Fearful>Calm_* = 2.80. The four-way interaction did not reach significance, *F*(28,2684)*_TimexBlockxStimulusxGroup_*= 0.67, *p*= .908 (see also Figure A6, A1-A3).

***Figure A5.*** Mean changes in tidal volume (ml) during 8s presentation of the CSs (conditioned stimulus) for each experimental group and phase. US-onset for the demonstrator (observational learning) and participants (direct acquisition) 8s after CS-onset. O1-O3: Observational learning, A1-A3: Direct acquisition phase, E1-E2: Direct extinction phase. O1-O3/ A1-A3: average across blocks of four trials. E1-E2: average across blocks of three trials. Error bars represent the standard error of the mean.

### Direct Extinction Phase

#### Respiratory Rate

During the first block of the extinction phase, respiratory rate increased in response to both CSs 3 – 8 s after onset, all *t*s> 3.73, all *p*s< .005, while respiratory rate did not change during CS-presentation in the second block of trials, *F*(7,1769.0)*_TimexBlock_*= 4.97, *p*< .001, *η_p_^2^*= .02, 95%CI [0.00, 1.00]. This pattern did not differ between stimuli or groups, all *F*s< 0.46, all *p*s> .951. Although we found a significant three-way interaction, *F*(2,1769.0)*_BlockxStimulusxGroup_*= 3.61, *p*= .027, *η_p_^2^*< .01, 95%CI [0.00, 1.00], this interaction was not driven by systematic changes in respiratory rate in response to CS+ relative to CS-, all *F*s*_Stimulus_*< 0.14, all *p*s> .707, and all *F*s*_StimulusxGroup_* < 1.18, all *p*s> .313, but resulted participants of the control condition, *F*(1,1769)*_BlockxStimulus_*= 5.74, *p*= .017, who exhibited an increase in respiratory rate in response to CS- during the first block of trials, while respiratory rate was relatively reduced in response to CS- during the second block of trials, *t*(70.2)*_E1>E2_*= 3.04, *p*= .003 (see Figure A4, E1-E2). The four-way interaction did not reach significance, *F*(2,1769.0)*_TimexBlockxStimulusxGroup_*= 0.44, *p*= .962.

#### Tidal Volume

Tidal volume increased during CS-presentation, 7 – 8 s after CS-onset in the first block of trials in the extinction phase, all *t*s> 3.43, all *p*s< .014, and 5 – 8 s after CS-onset during the second block of trials, all *t*s> 3.65, all *p*s< .007, *F*(7,1769.0)*_Time x Block_* = 2.57, *p*= .012, *η_p_^2^*< .01, 95%CI [0.00, 1.00], which did not differ between stimuli or groups, all *F*s< 1.26, all *p*s> .267 (see Figure A5, E1-E2). Moreover, we found a significant three-way interaction, *F*(2, 1769.0)*_BlockxStimulusxGroup_*= 5.12, *p*= .006, *η_p_^2^*< .01, 95%CI [0.00, 1.00] , that was driven by observers of the fearful demonstrator, who showed higher tidal volume in response to CS- as compared to CS+, *t*(89.5)*_CS+>CS-_*= -2.85, *p*= .005, while changes in tidal volume did not further differentiate between CS+ and CS- for participants of all group, all *t*s*_CS+>CS-_* < 1.05, all *p*s> .298. The four-way interaction did not reach significance, *F*(14, 1769.0)*_TimexBlockxStimulusxGroup_*= 0.16, *p*= .999.

#### Minute Ventilation

Minute ventilation increased relative to baseline during CS-presentation 4 – 8 s after CS-onset for observers of the fearful demonstrator, all *t*s> 3.89, all *p*s< .003, and 5 – 8s s after CS-onset for observers of the calm demonstrator and participants of the control condition, all *t*s> 3.48, all *p*s< .012, *F*(14,1768)*_TimexGroup_*= 2.08, *p*= .010, *η_p_^2^*= .01, 95%CI [0.00, 1.00]. Moreover, a significant three-way interaction emerged, *F*(2,1769)*_BlockxStimulusxGroup_*= 5.12, *p*= .006, *η_p_^2^*< 0.01, 95%CI [0.00, 1.00], that was driven by observers of the fearful demonstrator, who showed higher minute ventilation in response to CS- relative to CS+ during the first block of the extinction phase*, t*(87.8)*_CS+>CS-_*= -3.22, *p*= .002, while changes in minute ventilation did not differentiate between CS+ and CS- for observers of the calm demonstrator or participants of the control condition, all *F*s*_Stimulus_*< 3.31, all *p*s> .074, and all *F*s*_BlockxStimulus_*< 3.65, all *p*s> .056, The four-way interaction did not reach significance, *F*(14,1769)*_TimexBlockxStimulusxGroup_*= 0.16, *p*= .999 (see also Figure A6, E1-E2).

***Figure A6.*** Mean changes in minute ventilation (liters per minute) during 8s presentation of the CSs (conditioned stimulus) for each experimental group and phase. US-onset for the demonstrator (observational learning) and participants (direct acquisition) 8s after CS-onset. O1-O3: Observational learning, A1-A3: Direct acquisition phase, E1-E2: Direct extinction phase. O1-O3/ A1-A3: average across blocks of four trials. E1-E2: average across blocks of three trials. Error bars represent the standard error of the mean.

| **Table S3.** Overview of the Statistical Significance for Complementary Analyses of Cardiac and Respiratory Measures | | | | | |
| --- | --- | --- | --- | --- | --- |
| **Exp. Phase** | **Measure** | ***F* Test** | ***F(df)*** | ***p*** | ***η_p_^2^*** |
| Observational Learning Phase | | | | | |
|  | D1 | Block x Stimulus x Group | 0.36 (4,320) | .835 | <.01 |
|  | A | Block  Block x Stimulus x Group | 6.83 (2,320)  1.16 (4,320) | .001**  .329 | .04  .01 |
|  | D2 | Block  Block x Stimulus x Group | 6.75 (2,320)  0.24 (4,320) | .001**  .918 | .04  <.01 |
|  | BF | Block x Stimulus x Group  Time x Stimulus x Block x Group | 3.89 (4,2745)  0.54 (28,2745) | .004*  .978 | <.01  <.01 |
|  | TV | Time  Block x Stimulus x Group  Time x Stimulus x Block x Group | 6.02 (7,2684)  2.80 (4,2684)  0.36 (28,2684) | <.001***  .025*  .999 | .02  <.01  <.01 |
|  | MV | Time  Block x Stimulus x Group  Time x Stimulus x Block x Group | 16.89 (7,2684)  2.53 (4,2684)  0.62 (28,2684) | <.001***  .039*  .943 | .04  <.01  <.01 |
| Direct Acquisition Phase | | | | | |
|  | D1 | Block x Stimulus x Group | 2.51 (4,315.0) | .042*** | .03 |
|  | A | Block x Stimulus x Group | 0.53 (4,315.8) | .718 | <.01 |
|  | D2 | Stimulus  Block x Stimulus x Group | 11.11 (1,316.0)  1.25 (4,315.4) | <.001***  .289 | .03  .02 |
|  | BF | Time x Stimulus  Block x Stimulus  Time x Block x Stimulus x Group | 4.65 (7,2684)  11.23 (2,2684)  0.36 (28,2684) | <.001***  <.001***  .999 | .01  <.01  <.01 |
|  | TV | Time  Block x Stimulus x Group  Time x Stimulus x Block x Group | 30.62 (7,2684)  3.11 (4,2684)  0.98 (28,2684) | <.001***  .014*  .498 | .07  <.01  .01 |
|  | MV | Time  Block x Stimulus x Group  Time x Stimulus x Block x Group | 16.87 (7,2684)  2.53 (4,2684)  0.62 (28,2684) | <.001***  .039*  .943 | .23  <.01  <.01 |
| Direct Extinction Phase | | | | | |
|  | D1 | Block x Stimulus x Group | 3.27 (2,120.0) | .042*** | .05 |
|  | A | Block x Stimulus x Group | 0.09 (2,55.8) | .917 | .04 |
|  | D2 | Block x Group  Block x Stimulus x Group | 4.49 (2,62.5)  0.22 (2, 122.4) | .015*  .800 | .13  <.01 |
|  | BF | Time x Block  Block x Stimulus x Group  Time x Block x Stimulus x Group | 4.97 (7,1768)  3.61 (2,1769)  0.44 (14,1769) | <.001***  .027*  .962 | .02  <.01  <.01 |
|  | TV | Time x Block  Block x Stimulus x Group  Time x Block x Stimulus x Group | 2.57 (7,1768)  8.91 (2,1769)  0.21 (14,1769) | .012*  <.001***  .999 | .01  <.01  <.01 |
|  | MV | Time x Group  Block x Stimulus x Group  Time x Block x Stimulus x Group | 2.08 (14,1769)  5.12 (2,1769)  0.16 (14,1769) | .010*  .006**  .999 | .02  <.01  <.01 |
| *Note.* Significant effects are reported in addition to the relevant three-way and four-way interactions. D1= initial cardiac deceleration, A= cardiac acceleration, D2= late cardiac deceleration. BF= changes in respiratory rate, TV= changes in tidal volume, MV= changes in minute ventilation. *p < .05. **p < .01. ***p < .001. | | | | | |

References

[1]	Szczepanik M, Kazmierowska AM, Michalowski JM, Wypych M, Olsson A, Knapska E. Observational learning of fear in real time procedure. Sci Rep 2020;10(1). https://doi.org/10.1038/s41598-020-74113-w.

[2]	Benke C, Hamm AO, Pané-Farré CA. When dyspnea gets worse: Suffocation fear and the dynamics of defensive respiratory responses to increasing interoceptive threat. Psychophysiology 2017;54(9):1266–83. https://doi.org/10.1111/psyp.12881.

[3]	Alcan E, Benke C, Melzig CA. A threat from within: Learning to fear by observing aversive bodily symptoms in others. Behav Res Ther 2024;178:104555. https://doi.org/10.1016/j.brat.2024.104555.

[4]	Radomsky AS, Rachman S, Thordarson DS, McIsaac HK, Teachman BA. The Claustrophobia Questionnaire. J Anxiety Disord 2001;15(4):287–97. https://doi.org/10.1016/S0887-6185(01)00064-0.

[5]	Taylor S, Zvolensky MJ, Cox BJ, Deacon B, Heimberg RG, Ledley DR et al. Robust dimensions of anxiety sensitivity: development and initial validation of the Anxiety Sensitivity Index-3. Psychol Assess 2007;19(2):176–88. https://doi.org/10.1037/1040-3590.19.2.176.

[6]	Ehlers A. Somatic symptoms and panic attacks: a retrospective study of learning experiences. Behaviour Research and Therapy 1993;31(3):269–78. https://doi.org/10.1016/0005-7967(93)90025-P.

[7]	Globisch, J., Hamm, A., SChneider, R., & Vaitl, D. A computer program for scoring reflex eyeblink and electrodermal responses written in Pascal 1993;30(1).

[8]	Gatchel RJ, Lang PJ. Accuracy of psychophysical judgments and physiological response amplitude. Journal of Experimental Psychology 1973;98(1):175–83. https://doi.org/10.1037/h0034312.

[9]	Mueller EM, Sperl MFJ, Panitz C. Aversive Imagery Causes De Novo Fear Conditioning. Psychol Sci 2019;30(7):1001–15. https://doi.org/10.1177/0956797619842261.
